# Selective Targeting of *CTNNB1-*, *KRAS-* or *MYC-*Driven Cell Growth by Combinations of Existing Drugs

**DOI:** 10.1371/journal.pone.0125021

**Published:** 2015-05-27

**Authors:** Joost C. M. Uitdehaag, Jeroen A. D. M. de Roos, Antoon M. van Doornmalen, Martine B. W. Prinsen, Jill A. P. Spijkers-Hagelstein, Judith R. F. de Vetter, Jos de Man, Rogier C. Buijsman, Guido J. R. Zaman

**Affiliations:** Netherlands Translational Research Center B.V., Oss, The Netherlands; University of Queensland Diamantina Institute, AUSTRALIA

## Abstract

The aim of combination drug treatment in cancer therapy is to improve response rate and to decrease the probability of the development of drug resistance. Preferably, drug combinations are synergistic rather than additive, and, ideally, drug combinations work synergistically only in cancer cells and not in non-malignant cells. We have developed a workflow to identify such targeted synergies, and applied this approach to selectively inhibit the proliferation of cell lines with mutations in genes that are difficult to modulate with small molecules. The approach is based on curve shift analysis, which we demonstrate is a more robust method of determining synergy than combination matrix screening with Bliss-scoring. We show that the MEK inhibitor trametinib is more synergistic in combination with the BRAF inhibitor dabrafenib than with vemurafenib, another BRAF inhibitor. In addition, we show that the combination of MEK and BRAF inhibitors is synergistic in *BRAF*-mutant melanoma cells, and additive or antagonistic in, respectively, *BRAF*-wild type melanoma cells and non-malignant fibroblasts. This combination exemplifies that synergistic action of drugs can depend on cancer genotype. Next, we used curve shift analysis to identify new drug combinations that specifically inhibit cancer cell proliferation driven by difficult-to-drug cancer genes. Combination studies were performed with compounds that as single agents showed preference for inhibition of cancer cells with mutations in either the *CTNNB1* gene (coding for β-catenin), *KRAS*, or cancer cells expressing increased copy numbers of *MYC*. We demonstrate that the Wnt-pathway inhibitor ICG-001 and trametinib acted synergistically in Wnt-pathway-mutant cell lines. The ERBB2 inhibitor TAK-165 was synergistic with trametinib in *KRAS*-mutant cell lines. The EGFR/ERBB2 inhibitor neratinib acted synergistically with the spindle poison docetaxel and with the Aurora kinase inhibitor GSK-1070916 in cell lines with *MYC* amplification. Our approach can therefore efficiently discover novel drug combinations that selectively target cancer genes.

## Introduction

The aim of combination drug treatment in cancer therapy is to achieve improved response rates and to decrease the probability of the development of drug resistance [1–3]. The discovery of new effective drug combinations is, however, constrained by the costs of carrying out systematic combination studies in the clinic and by the large number of possible drug combinations [4–6]. Cancer cell lines are an attractive model to investigate new drug combinations because they can be used to determine whether new combinations are truly synergistic, as opposed to additive [7, 8]. Moreover, cancer cell lines provide a good representation of the diversity of genetic changes that drive human cancers [9, 10].

In the past three decades the molecular causes of most of the major cancers have been identified, and this has led to the development of a number of medicines that target specific signaling pathways that are perturbed in cancer. Examples are imatinib, targeting a specific fusion protein of ABL kinase in chronic myeloid leukemia [11], and vemurafenib and dabrafenib, targeting a mutant form of the protein kinase BRAF in metastatic melanoma [12, 13]. These targeted therapies bring great benefit to patients, because they improve survival rates with less side effects than traditional, less selective, cytotoxic drugs. However, available targeted therapies are only beneficial to a small fraction of cancer patients, while after an initial good response, drug resistance often develops, similar to treatment with cytotoxic agents [14]. Furthermore, for some of the most frequently occurring oncogenic drivers, such as β-catenin (encoded by the gene *CTNNB1)*, KRAS and MYC, it has been shown to be difficult to identify small molecules that modulate these targets specifically, and these targets are therefore referred to as ‘undruggable’ [15].

Targeting of cell growth driven by undruggable cancer genes has been shown by RNA interference (RNAi)-based screenings. Knock-down of downstream, parallel, or compensatory mechanisms can lead to the selective killing of cells that express mutant *CTNNB1*, *KRAS*, or overexpress *MYC* [16–24]. However, attempts to translate these ‘synthetic-lethal’ studies to drug therapy have largely failed due to lack of efficacy (compare, *e*.*g*., references [19] and [25]). Potentially, better responses could be obtained by inhibiting multiple pathways downstream of the cancer gene, which is often a central signaling node. Such an approach of dual inhibition has never been tested, primarily because it is difficult to make a dose-range of inhibition of target activity with RNAi, and to apply a knock-down on two targets simultaneously. Pharmacological interference has the potential to resolve these problems, because it is feasible to target multiple pathways, and because results can be directly translated to relevant in *vivo* efficacy models [26].

There are some exciting examples of synergistic drug combinations involving targeted inhibitors. For instance, Liu *et al*. showed that inhibition of the MAPK pathway with MEK inhibitors acts synergistically with inhibition of the PI3K pathway [27]. Investigators from GlaxoSmithKline showed that the MEK inhibitor trametinib acts synergistically with the BRAF inhibitor dabrafenib, although both inhibitors act in the same pathway [28]. The combination of the two drugs is approved by the U.S. Food and Drug Administration (FDA) for the treatment of *BRAF*-mutant metastatic melanoma. A third combination of targeted inhibitors are BRAF and EGFR inhibitors in *BRAF*-mutant colorectal cancer. The BRAF inhibitor vemurafenib lacks efficacy in this cancer, due to a feedback regulatory mechanism resulting in high EGFR activity [29,30]. This feedback mechanism can be suppressed by combination with EGFR inhibitors [29,30]. All three examples show that synergistic effects between targeted drugs are possible, whether they inhibit different, or similar pathways.

It is important to consider that the signaling pathways that are inhibited by targeted drugs, such as the PI3K, MEK, BRAF and EGFR pathways, are also essential for proliferation of normal, non-transformed cells. For this reason, many targeted inhibitors are also toxic to cells that do not express specific oncogenes. If these toxicities would also synergize, the combination of two compounds would not result in an increased therapeutic window. On the other hand, if a combination would work exclusively synergistic in a specific tumor cell population (*e*.*g*., *BRAF*-mutant cells) and not in other cells (*i*.*e*., *BRAF*-wild type, or non-malignant cells), this would amplify the specific effect of the inhibitors. In parallel with the term ‘targeted’ for single agents, these combinations could be called ‘targeted combinations.’

Here, we present novel drug combinations, specifically targeted at Wnt-pathway, *KRAS* or *MYC*-dependent cell growth. The finding is based on evaluating synthetic lethal effects after screening of a drug library in a panel of forty-four cancer cell lines. More than hundred new and established drugs, clinical and pre-clinical compounds have been characterized on this panel, which is being used to identify novel genomic drug sensitivity markers for anti-cancer drugs [31]. We have used this data set to select compounds that specifically inhibit cell lines carrying undruggable oncogenic drivers. A subsequent study of the synergistic interactions between these cellularly selective single agents, resulted in several novel drug combinations that target these ‘difficult-to-drug’ cancer genes. This demonstrates that it is possible to more optimally exploit the vulnerabilities of cancer cells by drug combinations, thereby increasing the efficacy of targeted therapy.

## Materials and Methods

### Compounds

All reference inhibitors and cytostatic agents were obtained from commercial vendors (overview in S1 Table). Dry powders of reference compounds were stored as indicated by the supplier. Before testing, compounds were dissolved in dimethyl sulfoxide (DMSO) and diluted in 20 mM sterile Hepes buffer pH 7.4, before addition to microtiter plates with cells [31].

### Cell proliferation assays

All cell lines were purchased from the American Type Culture Collection (ATCC) (Manassas, VA, U.S.A.) (S2 Table) and cultured in the media as recommended by ATCC. All cells used were within nine passages of the original ATCC vial. Proliferation assays were carried out as described before [31] in 384-well plates with incubation with compound for 72 hours. Cell number was optimized for each cell line, to maximize assay window and to ascertain that growth was not limited by cell density. Compound effects were measured in a 9-point dilution series in duplicate. The final DMSO concentration during incubation was 0.4% (v/v) in all wells. As readout, we determined intracellular ATP content as an indirect measure of cell number, using ATPlite 1 Step solution (Perkin Elmer, Groningen, The Netherlands). The effect of the compounds on cell growth was calculated relative to control wells containing only 0.4% (v/v) DMSO. IC_50_s were fitted by non-linear regression using XLfit5. Maximum and minimal signals were locked, where appropriate, to obtain the best fit as indicated by the *F*-test as implemented in XLfit5. If IC_50_s did not fit within the tested concentration range, compounds were retested after further dilution.

### Curve shift synergy experiments

**Fig 1:**
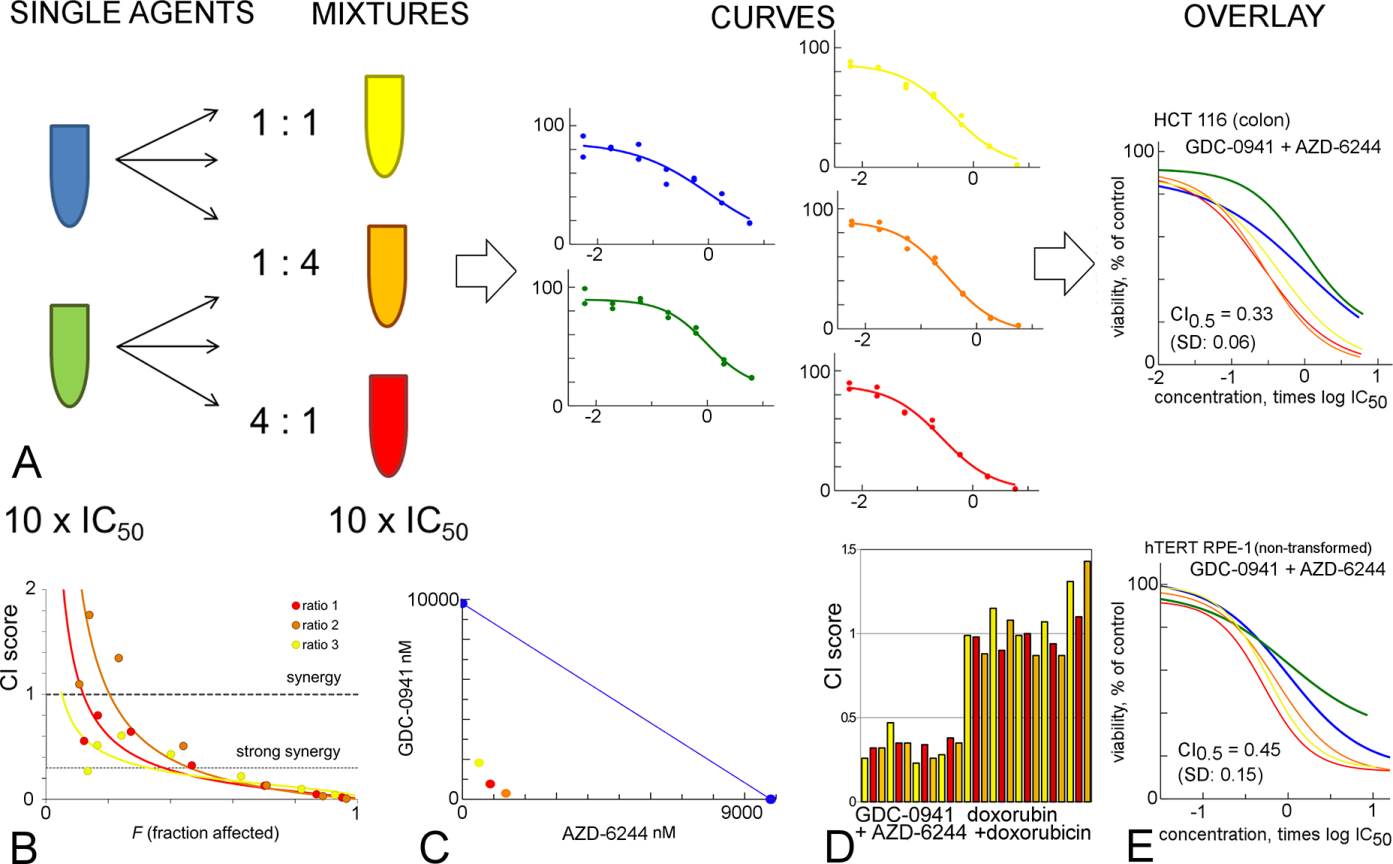
Setup of the curve shift synergy experiments. **A**: First, concentrated mixtures are made of the single agents. Subsequently, dose response curves are generated. For the overlay, all concentrations are scaled relative to the measured IC_50_s of the single agents. The example shown is the effect of the PI3-kinase inhibitor GDC-0941 (green) and the MEK inhibitor AZD-6244 (blue) on proliferation of the HCT 116 cell line. Mixture ratios used were 1:1, red; 4:1, orange; 1:4, yellow. **B**: Calculation of the Combination Index (CI). The fraction affected *(F)* is equivalent to 1/100 of the %-effect. If CI < 1, compounds show synergy. The fitted CIs at *F* = 0.5 (50% effect), for all mixtures, are reported as CI_0.5_. **C**: Calculation of the isobologram [7]. Single agent concentrations needed to achieve 75% effect in the cell proliferation assay are displayed in blue dots and connected by the blue line. The concentrations where the combination curves achieve 75% growth effect are displayed in red, yellow and orange, where the x and y coordinates are the respective component concentrations. If the combination points lie below the blue line, there is synergy. **D**: Reproducibility of CI_0.5_ measurements in a positive control of AZD-6244 / GDC-0941 (light bars, average 0.33, SD: 0.06, n = 12) and a negative control of doxorubicin / doxorubicin (dark bars, average 1.04, SD: 0.16, n = 15). Both were combined in the HCT 116 cell proliferation assay. Every bar represents an individual mixture ratio (yellow, red, orange) that was tested in duplicate. **E:** Curve shift experiment of the AZD-6244 / GDC-0941 combination in the RPE-1 fibroblast cell line, which is immortalized using hTERT. Here, the combination also shows synergy.

For all synergy experiments, cell line proliferation assays were performed as outlined above. For curve shift analysis, compound stocks were diluted in DMSO on the day of the experiment, to concentrations of 3160 times their IC_50_, as determined in the single agent experiments (S3 Table). Both solutions were subsequently mixed in three volume ratios (1:1, 1:4 and 4:1, unless otherwise indicated, Fig 1) [8, 32]. Because of the IC_50_ matching, all stock solutions and mixtures will be equipotent when there is no synergy or antagonism between the two agents tested. The stocks of the mixtures and single agents were further diluted in DMSO to generate a 7-points dose-response series in duplicate. The final DMSO concentration during incubation was 0.4% (v/v) in all wells. Final assay concentrations range, for the single agents, between 10 and 0.01 times their IC_50_ (10 and 0.01 IC_50_ equivalents). For the single agent curves, IC_50_s were fitted on the %-effect data by non-linear regression using XLfit5. To correct for inter-assay variation, the IC_50_s of the single agents in the synergy experiment were used to calculate the total mixture potencies. If, for instance, in the final synergy experiment a single agent potency was 1.2 times higher than expected, all x-axis values along the dilution curve were divided by 1.2 (Fig 1A). This ensures proper overlay of all curves in the experiment.

**Fig 2:**
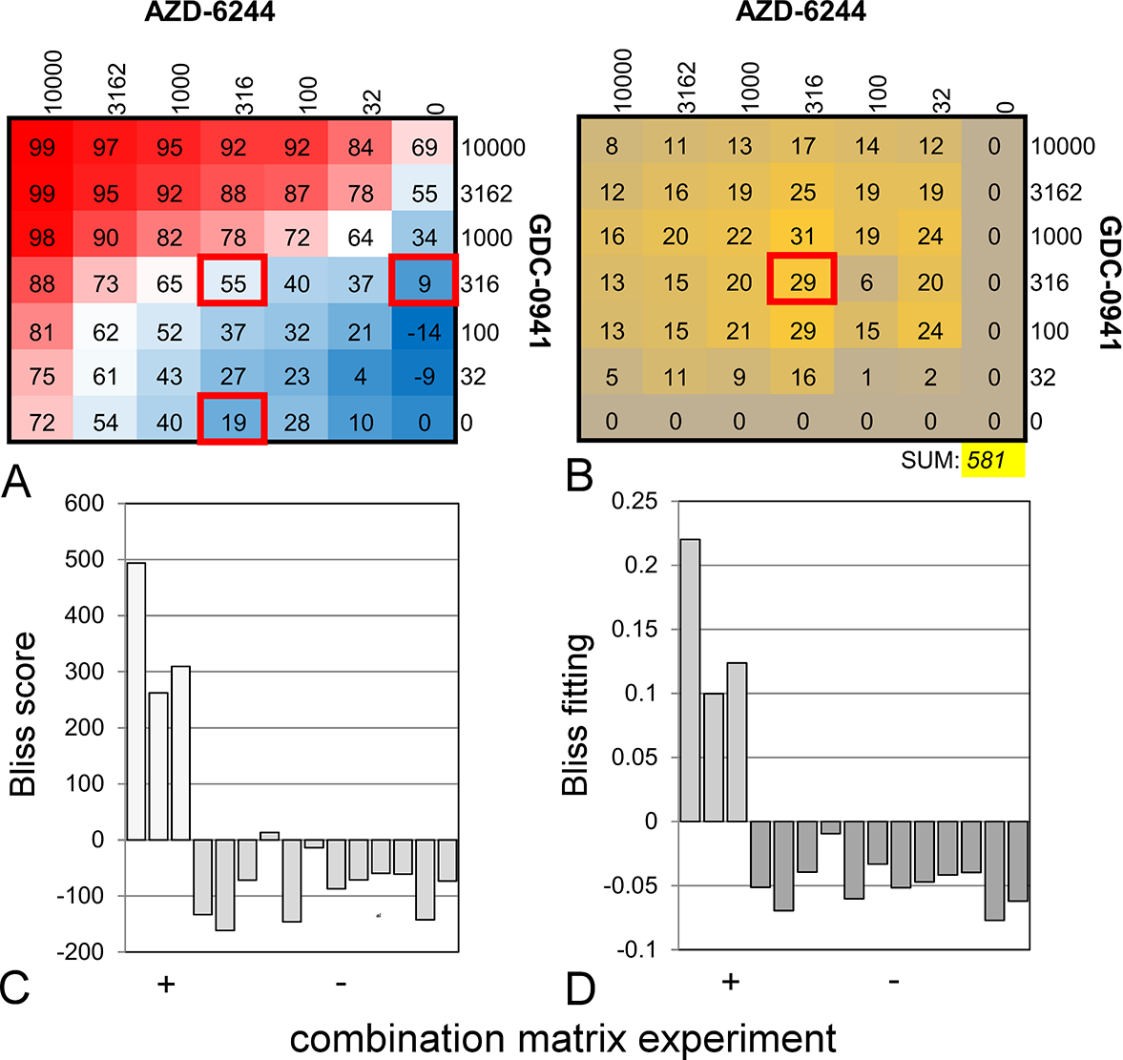
Setup of a combination matrix experiment, which is an alternative to a curve shift experiment. Dilution series of the MEK inhibitor AZD-6244 (rows, in nM) and the PI3K inhibitor GDC-0941 (columns, in nM) were mixed and their effect was measured in the HCT 116 cell line proliferation assay. **A**: Relative inhibitory effects (%) in the assay. **B**: Bliss scores calculated by relating the mixture effects to the single agent effects, using the following procedure, illustrated by the data in red squares: 1. the %-inhibition effects are converted to fractional growth effects by 1 - (%-inhibition / 100). For the indicated squares, the values become 0.45, 0.91 and 0.81, respectively; 2. The Bliss score is then equal to the observed effect for the mixture, with the additive effects of the single agents subtracted. This amounts to 0.45 - (0.91 · 0.81) = 0.29; 3. This leads to the value of 29% indicated in the right panel. The total Bliss score in yellow shading is the summation of all wells [4, 33]. **C**: Reproducibility of the combination matrix screening. The experiment was carried out using the same reagents as in Fig 1D. Positive controls are in white (average 355, SD: 122, n = 3), negatives in grey (average -84, SD: 54, n = 12). Bliss scoring was performed as outlined under panel B. **D**: as panel C, where the outcome is evaluated with a Bliss parameter fitting protocol to smoothen assay noise [5] (S1 Equation, positive controls in light grey (average 0.14, SD: 0.06, n = 3), negatives in dark grey (average -0.05, SD: 0.02, n = 12).

**Fig 3:**
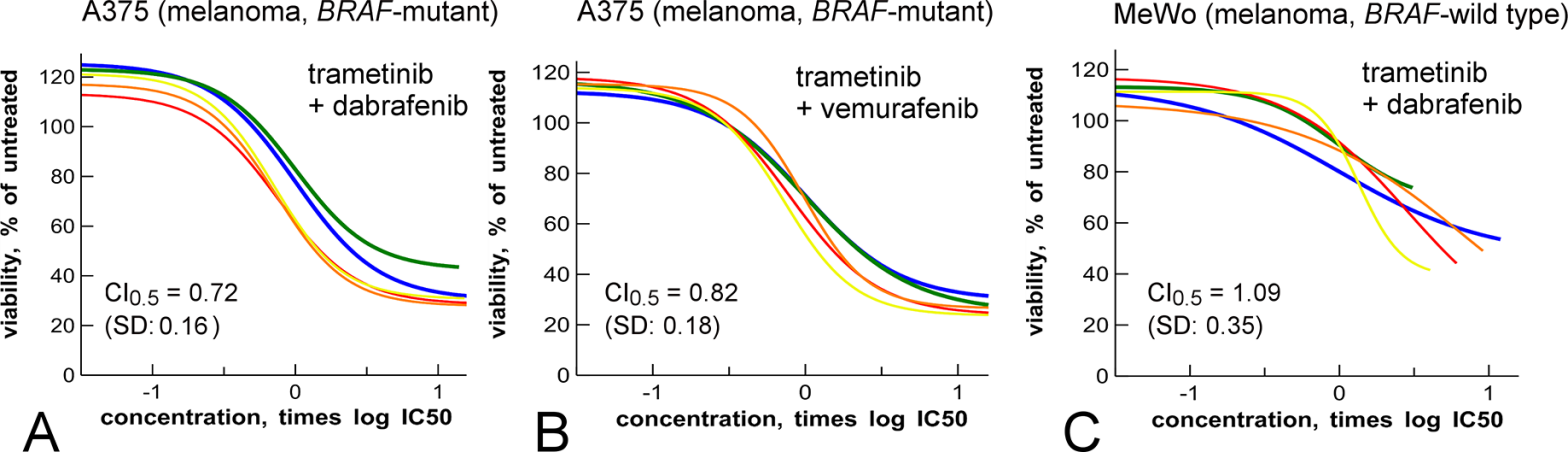
Targeted synergy between MEK- and BRAF-inhibitors. **A**: Curve shift experiment of trametinib (blue) and dabrafenib (green) in a proliferation assay of the *BRAF*-mutant A375 cell line. Yellow, red, orange are mixture curves as described in Fig 1. **B**: as panel A but with vemurafenib (green) as BRAF inhibitor. **C**: As panel A, but using proliferation assay of the *BRAF*-wild type melanoma MeWo cell line. Note that in panels A and B, there is synergy, although more in A than in B. In panel C, there is no synergy, as judged by CI and efficacy. The high variation for the CI in panel C is caused by the partial efficacy of all curves.

**Fig 4:**
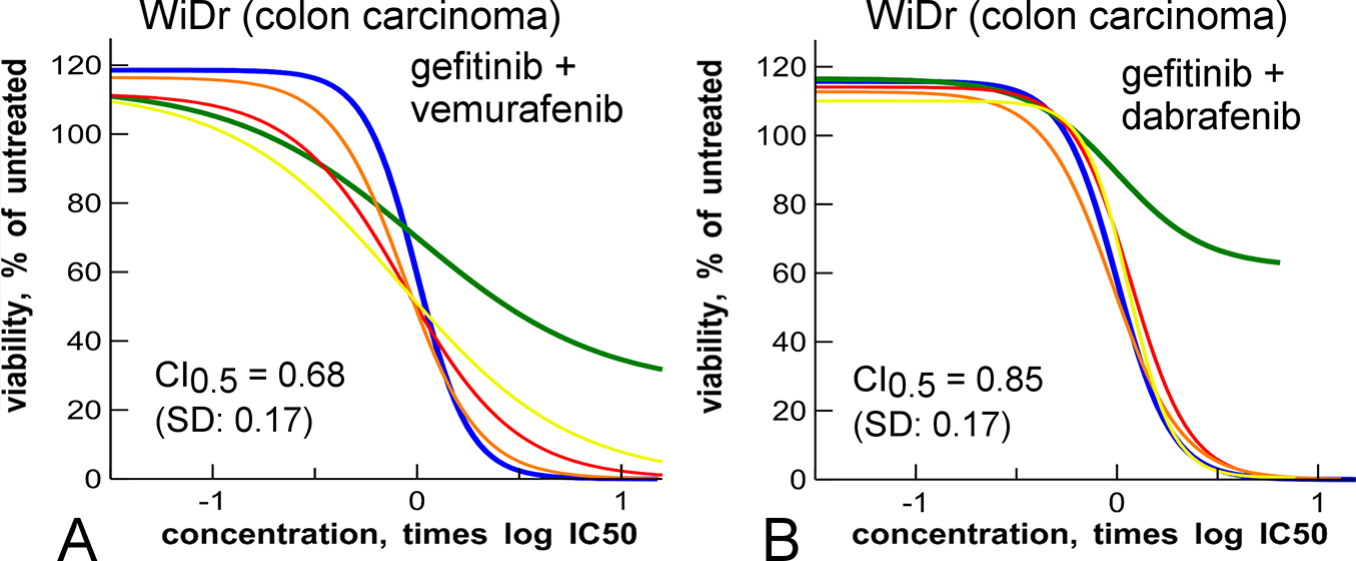
Synergy between EGFR and BRAF inhibitors in a colon carcinoma line. **A**: Curve shift experiment of the EGFR inhibitor gefitinib (blue) and the BRAF inhibitor dabrafenib (green) in a proliferation assay of the *BRAF*-mutant WiDr colon carcinoma cell line. Yellow, red, orange are mixture curves as described in Fig 1. **B**: as panel A but with vemurafenib (green) as BRAF inhibitor. In panel A, there is clear synergy between the two drugs, in panel B, there is less synergy.

**Fig 5:**
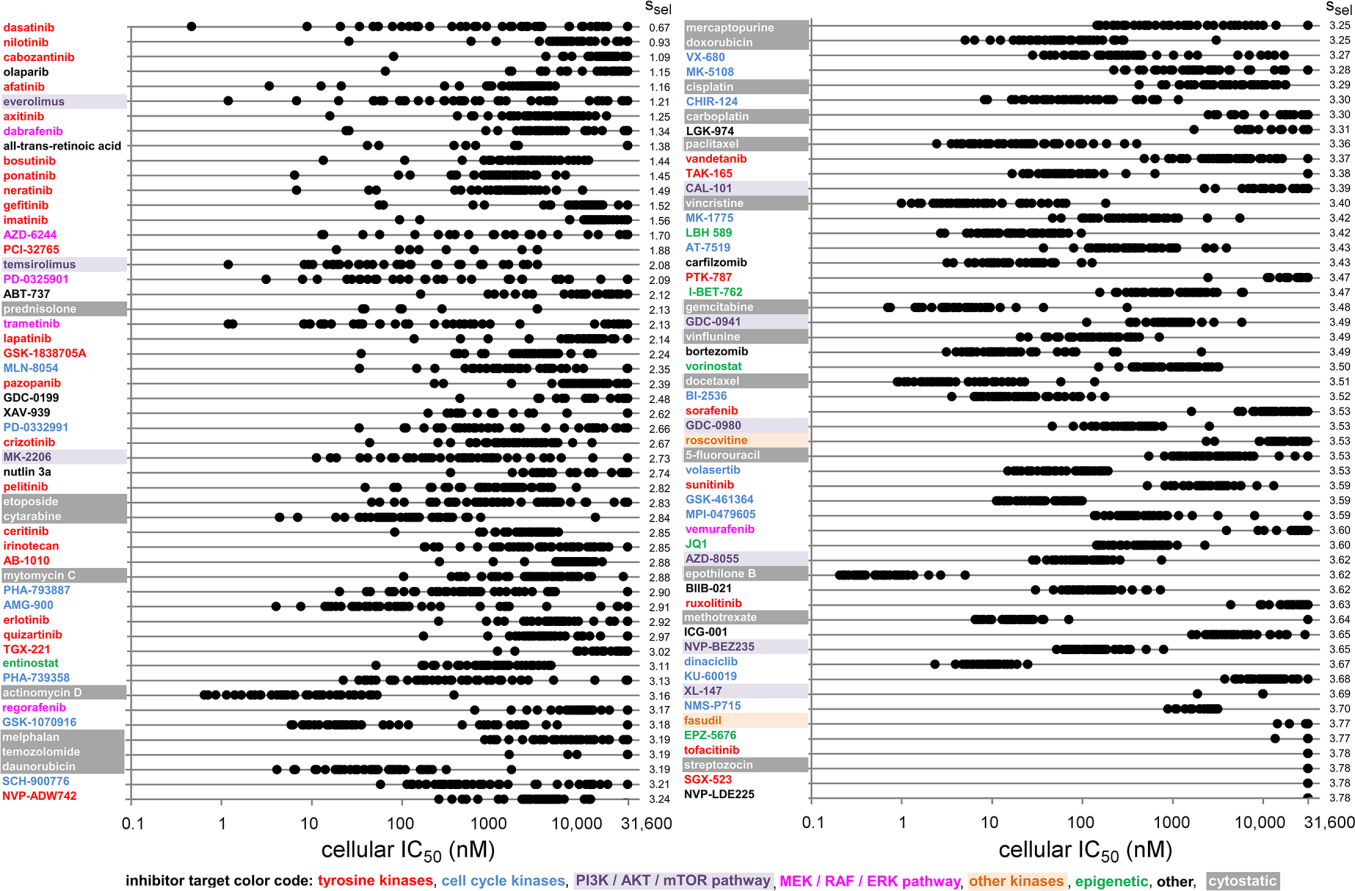
Distribution of IC_50_s after profiling of a drug library on a 44-cell line panel. Compound effects were measured in dose-response in parallel on forty-four different cell line proliferation assays. IC_50_s were fitted. Every black dot represents the IC_50_ of a separate compound and cell line combination. Each line contains forty-four IC_50_s. Compounds are ordered according to their cellular selectivity, as indicated by an entropy value (s_sel_) that is based on a Boltzmann-weighted average of IC_50_ values [34]. At the top-left are the most selective compounds in the cell proliferation assays, *i*.*e*., the compounds that gave the most differentiated effects in the proliferation assays across the 44-cell line panel. The least selective drugs, which gave the least differentiated effects across cell lines, are indicated bottom-right. Names of the inhibitors are colored according to their biological mechanism.

**Fig 6:**
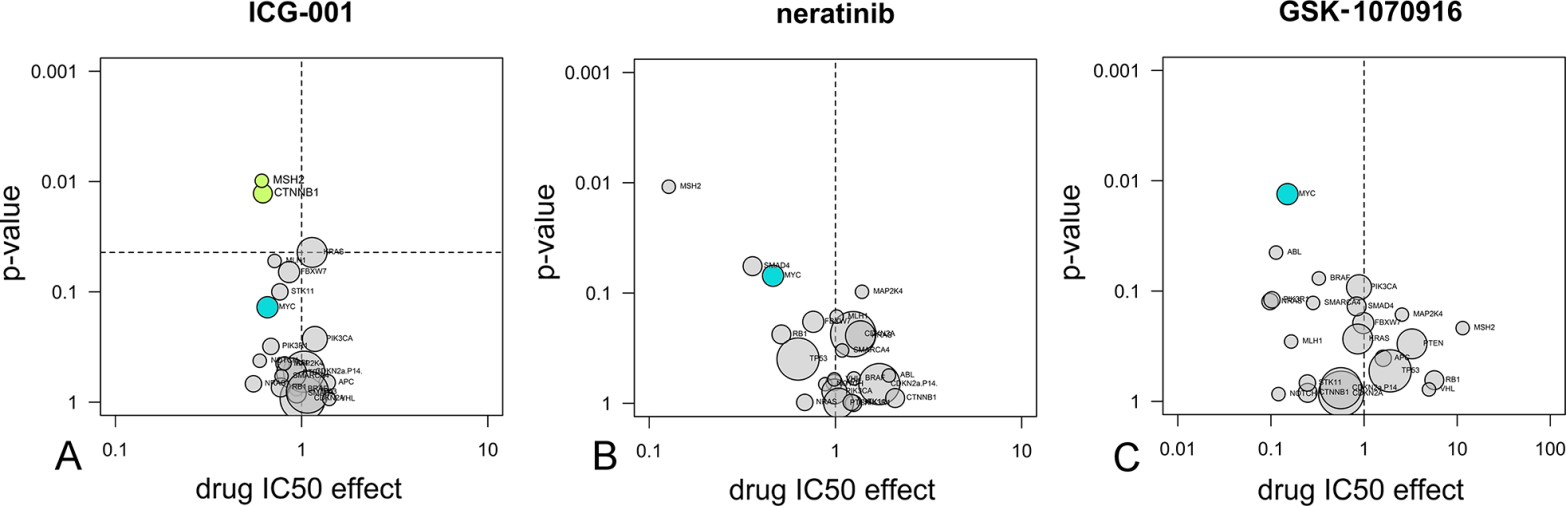
Anova analysis to reveal drug sensitivity markers. The sensitivity of drugs for twenty-three common cancer gene mutations was analysed statistically through Anova, based on the IC_50_ data on forty-four cell lines, and depicted in volcano plots. On the x-axis: IC_50_ shift between wild type and mutant cell lines. On the y-axis: p-value for significance in the Anova variance test. Significance was corrected for multiple-testing and all associations above the threshold (dotted line) were colored green [31]. In panels B and C no associations are above the multiple testing threshold and therefore no dotted line is shown. Areas of circles are proportional to the number of cell lines carrying mutations. **A**: The Wnt-pathway inhibitor ICG-001 [39] is relatively potent in cell lines containing *CTNNB1* or *MSH2* mutations. **B**: the EGFR inhibitor neratinib (HKI-272) [61] is relatively potent in cell lines containing *MSH1*, *SMAD4* mutations, or *MYC* amplifications (blue). **C**: the Aurora inhibitor GSK-1070916 [62] is relatively potent in cell lines containing *MYC* amplifications (blue) or *ABL* translocations.

**Fig 7:**
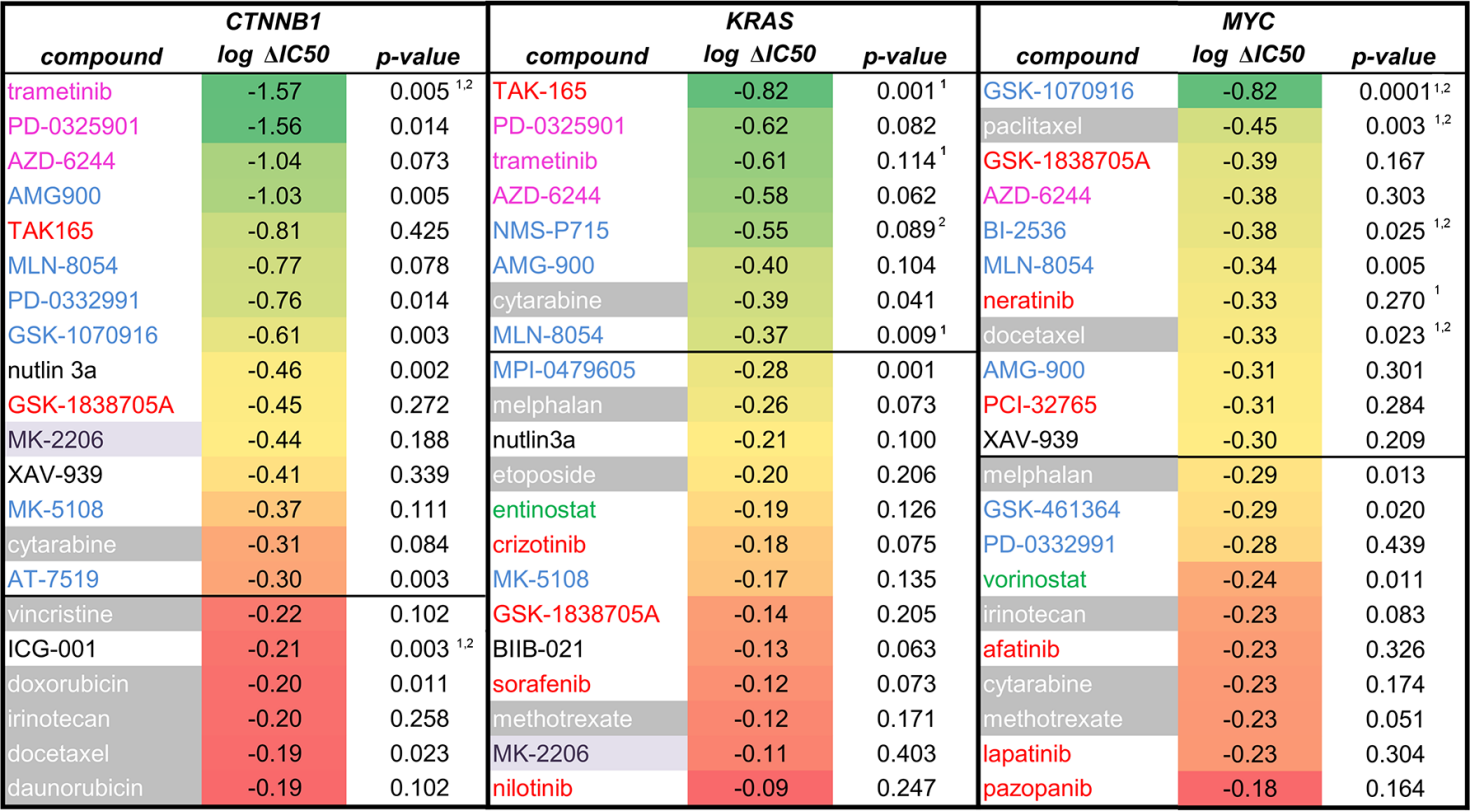
Compounds that most specifically inhibit *CTNNB1*- or *KRAS*-mutant, or *MYC*-amplified cell lines. Compound names were colored according to inhibitor class, analogous to Fig 5. **Δ log IC**_**50**_ indicates the ^10^log of the ratio between average IC_50_s in the wild type and mutant (amplified) cell lines. A horizontal line is drawn at -0.3, indicating a 10^–0.3^ = 0.501 shift in IC_50_, making the compound ^1^/_0.501_ ≈ 2 times more potent on mutant (amplified) cell lines. The forty-four cell line panel comprised four *CTNNB1*-mutants, ten *KRAS*-mutant and five *MYC*-amplified cell lines from a variety of tissue origins (see S2 Table). The **p-value** is calculated from a t-test, to validate if IC_50_s in mutant (amplified) cell lines are significantly lower than in wild type cell lines. ^1^compound selected for follow-up in synergy studies. ^2^ significant association in the drug sensitivity Anova analysis that tested 23 genes simultaneously.

**Fig 8:**
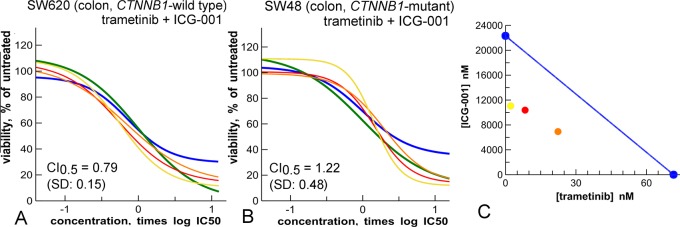
Synergy of the MEK inhibitor trametinib (blue) and the Wnt-pathway inhibitor ICG-001 (green) in colon cancer cell lines. **A:** Synergy in *CTNNB1*-wild type cell line SW620. This cell line is nevertheless a Wnt-pathway-mutant as it is mutated in *APC*. In addition, SW620 is *TP53*-mutant. **B:** Absence of synergy in the *CTNNB1*-mutant cell line SW48. This cell line is *TP53*-wild type**. C:** isobologram of results in panel A, at the 75% effect cut-off level. Yellow, red, orange are mixture curves as described in Fig 1.

**Fig 9:**
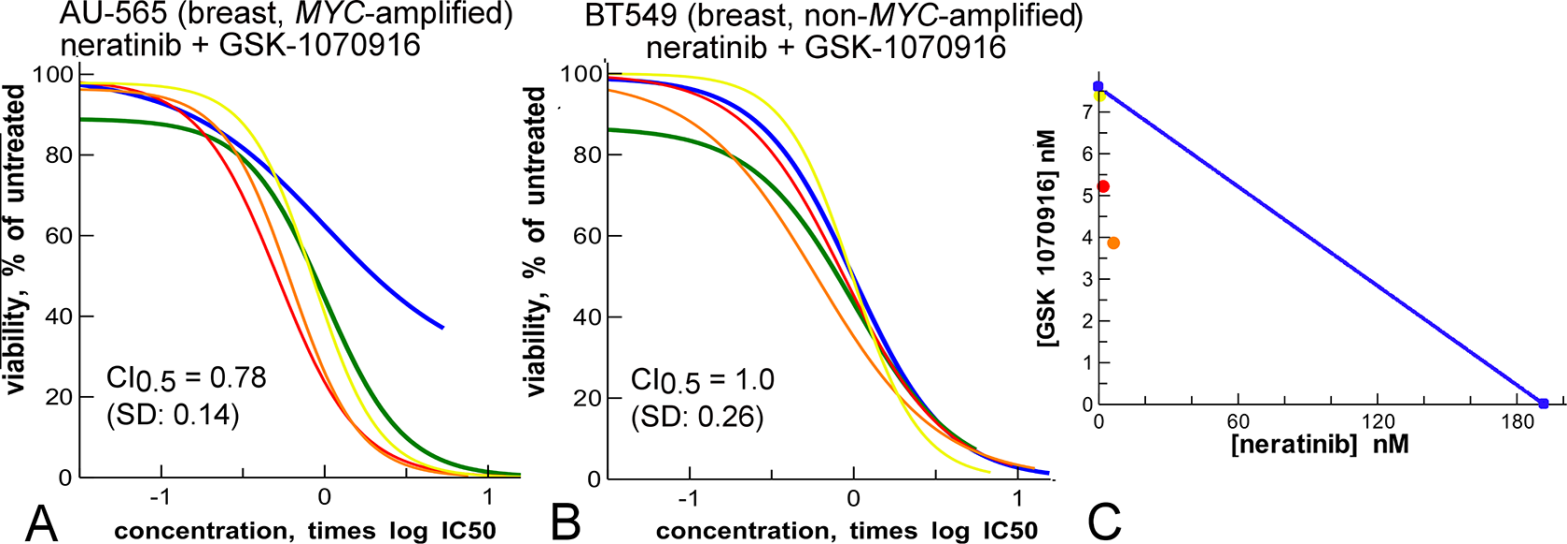
Synergy of neratinib (blue) and GSK-1070916 (green) depends on *MYC*-amplification. **A:** amplified cell line**. B:** non-amplified cell line**. C:** isobologram of results in panel A, at the 75% effect cut-off level. Yellow, red, orange are mixture curves as described in Fig 1. For panel B, 100% effect levels were set to the effect at the highest tested concentration.

**Fig 10:**
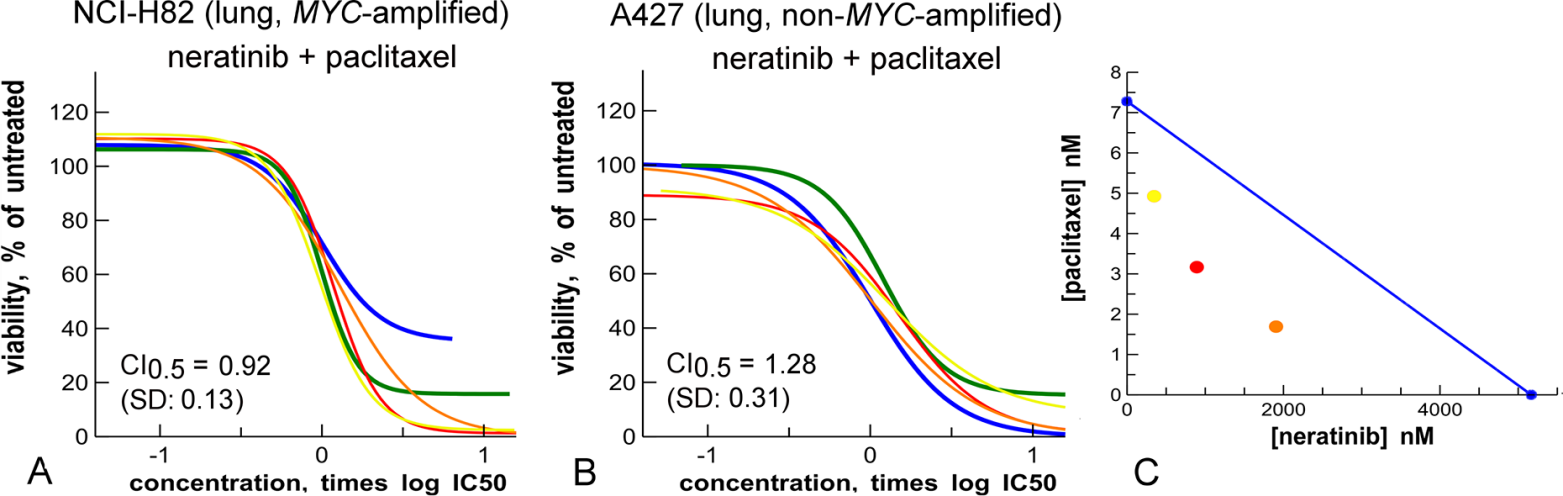
Synergy of neratinib (blue) and paclitaxel (green) in cells containing a *MYC* amplification. **A:** *MYC*-amplified. **B:** non-*MYC*-amplified. **C:** isobologram of results in panel A, at the 75% effect cut-off level. Yellow, red, orange are mixture curves as described in Fig 1.

**Fig 11:**
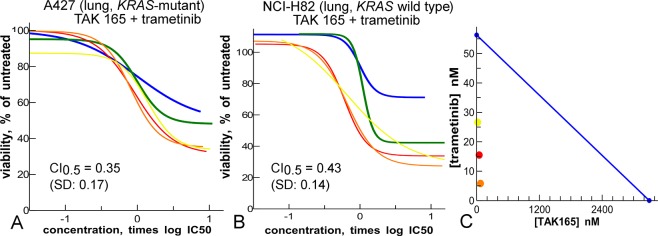
Synergy of the EGFR/ERBB2 inhibitor TAK-165 (blue) and the MEK inhibitor trametinib (green) is independent of *KRAS*-status. **A**: *KRAS*-mutant. **B**: *KRAS*-wild-type. **C:** isobologram of results in panel A, at the 50% effect cut-off level. Yellow, red, orange are mixture curves as in Fig 1.

From the same data, Combination Indices (CI) were determined according to the method of Chou and Talalay [8]. Briefly, from the fitted curve parameters, the concentrations of components A and B that induce 50% cell viability were determined, both for the single agent and mixtures experiments. Then, CI_0.5_, _mix1_ = [compound A]_mix1_ / [compound A]_single_ + [compound B]_mix1_ / [compound B]_single_. CI values were calculated in Microsoft Excel, using the same curve parameters that were used in evaluation of the curve shift. In cases with low efficacy curves, the program Calcusyn was used [8]. Because this software refits curves, it was not our method of preference. Each experiment contained three mixture ratios (1:1, 4:1, 1:4) in duplicate. Synergy experiments were repeated at least three times, on separate occasions, unless otherwise indicated (S4 Table). The three mixtures and three replicates (or more) resulted in at least nine independent CI_0.5_ values, from which averages and standard deviations were calculated. As further graphical check, isobolograms were generated in Microsoft Excel (*i*.*e*. Figs 1–11) [7].

### Combination matrix synergy experiments

To determine synergy with the combination matrix method [4–6], cell proliferation assays were performed as described above. At the day of the experiment, two single agents stocks were serially diluted in a 6-point series in either a row or a column (Fig 2A). A 7^th^ point was filled with 100% DMSO without compound. Subsequently, the complete 7 x 7 combination matrix was generated by 1:1 mixing of all single agent concentrations. Final tested compound concentrations range between 3 nM—1000 nM. The final DMSO concentration during incubation was 0.4% in all wells. The %-effect data from the proliferation assays were used to calculate an excess over Bliss additivity score, which was subsequently summed over the total combination matrix to generate a total Bliss score [4, 33] (Fig 2B). Alternatively, a Bliss model of compound independence was minimized on the %-effect data by adjusting a parameter β that is 1.0 for total additivity, and higher in case of synergy (S1 Equation) [5].

### Analysis of cell panel response data

The IC_50_ from the cell proliferation assays were used to calculate a selectivity parameter for each compound, the selectivity entropy (s_sel_) [34]. The s_sel_ was used to rank compounds for their cellular selectivity (Fig 5).

Anova analysis was used to determine whether there is a statistical correlation between drug sensitivity and the genotype of the cancer cell lines used in the proliferation assays. Cancer gene mutation status of the cell lines was downloaded from the Cancer Cell Line initiative (CCL, S2 Table) [9]. IC_50_ data were correlated to cell line genotype by type II Anova in the program R [35], Significance was evaluated after multiple testing correction of the p-values, and the results were depicted in volcano plots, according to a method outlined in earlier work [31] (Fig 6).

To evaluate if an inhibitor is significantly more potent in cell lines with particular genetic changes, a one-sided t-test was carried out in Microsoft Excel (Fig 7). This tested if IC_50_s in the group of oncogene-carrying cell lines were significantly lower than those in wild type cell lines (for cell line genotypes, see S2 Table, for individual IC_50_s, see S3 Table).

### Compound selection for combination studies

Based on the cell line response data, compounds were selected for combination testing. The underlying hypothesis was that compounds that work in similar oncogenic backgrounds, have a higher chance of forming synergistic pairs, providing these compounds have different biological mechanisms. High-ranking (in Fig 7) inhibitors of proliferative pathways such as RAS, EGFR or ERBB2 were first selected: trametinib for *CTNNB1*, TAK-165 for *KRAS*, neratinib for *MYC*. These were combined with high ranking inhibitors from other classes, *i*.*e*. cell cycle inhibitors or taxanes. Preference was given to partner compounds that showed significant association in Anova (p < 0.05) or that showed significance in the t-test (p < 0.05) combined with reasonable IC_50_ differences (logIC_50_ < -0.3, a factor 2). The selected compounds are shown in Table 1; individual IC_50_s in S3 Table. For *MYC*, the tyrosine kinase inhibitor neratinib was preferred over GSK-1838705A because neratinib is cellularly more active and selective. For *KRAS*, trametinib was chosen as additional partner to TAK-165, although p > 0.05, because it is known that MEK inhibitors are active in KRAS-mutant cell lines [36].

**Table 1:** Compounds selected for synergy studies.

genetic driver	compounds in combination set	biochemical target	IC_50_ shift	p-value^1^	p-value^2^
***BRAF***	Dabrafenib	BRAF kinase	284	1E-29	7E-6
**mutation**	Trametinib	MEK kinase	35	2E-8	0.2
	vemurafenib	BRAF kinase	3.0	0.2	4E-4
***CTNNB1***	Trametinib	MEK kinase	37.0	0.005	0.02
**mutation**	ICG-001	Wnt-pathway	1.6	0.003	0.01
***MYC***	neratinib	EGFR kinase	2.2	0.3	0.07
**amplification**	GSK-1070916	Aurora B/C kinase	6.6	0.0001	0.01
	BI-2536	PLK1 kinase	2.4	0.03	6E-3
	paclitaxel	spindle poison	2.8	0.003	0.008
	docetaxel	spindle poison	2.1	0.02	0.01
***KRAS***	TAK-165	ERBB2 kinase	6.6	0.001	0.3
**mutation**	trametinib	MEK kinase	4.1	0.1	0.9
	MLN-8054	Aurora A/B kinase	2.3	0.009	0.3

^1^Significance as determined by a one-gene t-test.

^2^Significance as determined by the 23-gene Anova analysis, before multiple testing correction. A p-value < 0.05 is considered statistically significant.

## Results

### Curve shift is the most robust setup to observe synergy

Much research has gone into defining the best way of determining synergy between two compounds in an *in vitro* assay. From this, two basic methods have evolved, the curve shift analysis (Fig 1) [8,32] and the combination matrix experiment with Bliss-scoring (Fig 2) [4–6]. We first performed robustness experiments to determine the optimal experimental setup.

For curve shift analysis, equipotent stock solutions of two drugs are mixed in various ratios and dose-response curves are determined. In case of synergy, the mixture curves are shifted leftward (Fig 1A). In case of antagonism, the mixture curves are shifted rightward. From the same experiment we calculated the Combination Index (CI) to quantify synergy (Fig 1B) [8], and visualized synergy in an isobologram (Fig 1C). The curve shift method can be used to determine which concentration is necessary to achieve a predetermined effect, *e*.*g*., 50% of maximum effect. This is known as the Loewe concept of synergy [37].

We validated the curve shift method by testing the combination of the PI3K inhibitor GDC-0941 and the MEK inhibitor AZD-6244 in colon carcinoma HCT 116 cells (Fig 1), a synergistic combination previously described by Haagensen *et al*. [38] following a biological discovery by Liu *et al*. [27]. The CI_0.5_ value of GDC-0941/AZD-6244 is 0.33, indicating strong synergy. In addition, the curve overlays show that at the highest concentrations, the combination also has increased efficacy compared to the single agents (Fig 1A).

To examine robustness, we repeatedly tested the GDC-0941/AZD-6244 combination, and the neutral combination of doxorubicin with doxorubicin (Fig 1D). The CI values of GDC-0941/AZD-6244 (average: 0.33, standard deviation (SD): 0.06, n = 12, Fig 1D) are well reproducible across experiments and across mixtures. The neutral combination has a CI of about 1, as theoretically expected (average: 1.04, SD: 0.16, n = 15, Fig 1D). This shows that the curve shift method can reliably detect synergy.

Next we evaluated the combination matrix method. Here, dilution series of two compounds are mixed along the rows and columns of a matrix (Fig 2A). The signal in each well is scored using the Bliss additivity criterion (Fig 2B) [4, 33]. Because changes in the relative (%) effect at predetermined compound concentrations are calculated, the Bliss approach is fundamentally different from the Loewe approach [37]. Also with the combination-matrix method, the combination of GDC-0941 and AZD-6244 showed synergy (Fig 2C). However, the variation between different experiments was significantly higher than in experiments where the curve shift method was used (average: 355, SD: 122, n = 3, Fig 2C). Because this variation could in part be due to absence of data fitting, we implemented an algorithm in which a synergy parameter β is fitted to the formula for Bliss additivity (see Methods and Fig 2D) [5]. This did not show better reproducibility (average: 0.14, SD: 0.06, n = 3, compare Figs 1D and 2D). Interestingly, the Bliss methods had the tendency to assign antagonism to the control of doxorubicin with itself.

Based on the data, we evaluated signal to noise ratios (average/SD) for the three synergy metrics. The GDC-0941/AZD-6244 combination experiment has a ratio of 5.5, 2.9 and 2.3 for curve shift, Bliss scoring and Bliss fitting methods, respectively. For the doxorubicin with doxorubicin control, these ratios are 6.5, 1.5 and 2.5, respectively. Both sets of values indicate that the curve shift has the best signal to noise ratio.

### Synergy between GDC-0941 and AZD-6244 in non-malignant cells

The PI3K-inhibitor GDC-0941 and the MEK inhibitor AZD-6244 are both targeted compounds in development for oncology, and show potent synergy (Fig 1A) However, since PI3K and MEK play a central role in regulating cell growth, these compounds also inhibit normal proliferating cells and thus exhibit significant toxicities [39]. If the synergy between the two compounds would be limited to malignant cell lines, this would provide new opportunities for their application as cancer therapy. For this reason we tested for synergy between GDC-0941 and AZD-6244 in two models of normal proliferating cells: human telomerase (hTERT)-immortalized cells derived from human retinal epithelia (hTERT RPE-1) and human foreskin fibroblasts (BJ-5ta) that also have been immortalized with hTERT. GDC-0941 inhibits the proliferation of these cell lines with IC_50_ of 760 nM and 1.1 μM, respectively. AZD-6244 inhibits their proliferation with IC_50_ of 91 and 94 nM. In the hTERT RPE-1 cell line, the combination works synergistically (CI_0.5_ = 0.45, SD: 0.15, n = 15, Fig 1E). Synergy was also found in the BJ-5ta cell line (CI_0.5_ = 0.43, SD: 0.09, n = 16, S1 Fig). This demonstrates that the synergy between GDC-0941 and AZD-6244 is not specific to cancer cells.

### Curve shift shows that trametinib and dabrafenib are a targeted drug combination

Next, we used the curve shift method to study the combination of the MEK inhibitor trametinib and the BRAF inhibitor dabrafenib, which has shown synergistic activity in *BRAF*-mutant melanoma cell lines [28], and in melanoma patients [40]. As single agents, dabrafenib and trametinib are potent inhibitors of the proliferation of the *BRAF*-mutant A375 melanoma cell line, with IC_50_ of 26 nM and 19 nM, respectively [31]. Curve shift assays confirmed synergy of the two compounds (Fig 3A). We also examined the combination of trametinib with vemurafenib, another BRAF inhibitor (Fig 3B). As single agent vemurafenib has an IC_50_ of 4 μM in the A375 cell line assay. Although the curve shift indicates that the combination of trametinib and vemurafenib is synergistic, the CI was higher (*i*.*e*., CI_0.5_ = 0.82, SD: 0.18, n = 15) than for the combination of trametinib and dabrafenib (CI_0.5_ = 0.72, SD: 0.16, n = 15, Fig 3).

Because dabrafenib and trametinib can also inhibit the growth of non-*BRAF*-transformed cells [31], we investigated if their synergy also occurs in the *BRAF*-wild type MeWo melanoma cell line. Both dabrafenib and trametinib inhibit the proliferation of this cell line with IC_50_s of 987 nM and 46 nM, respectively (Fig 3C). However, both compounds show only partial efficacy (Fig 3C), which is in line with the absence of an oncogenic *BRAF*-mutation in this cell line [9]. When mixed, the combination does not show synergy in the form of a curve shift or increased efficacy (CI_0.5_ = 1.09, SD: 0.35, n = 9, Fig 3C). In the non-transformed hTERT RPE-1 and BJ-5ta cell lines, the combination is even antagonistic (S2 Fig). From this, we conclude that the combination of dabrafenib and trametinib is specifically targeted to oncogenic BRAF-signaling.

### Curve shift analysis of EGFR and BRAF inhibition in a *BRAF*-mutant colon carcinoma line

Recently, it was suggested that the growth of *BRAF*-mutant colon carcinoma cell lines can be inhibited by combining a BRAF inhibitor with an EGFR inhibitor, which both were suggested to be inactive as single agents [29]. As this seemed an interesting example of a synergistic interaction that only takes place in a particular cancer cell type (*i*.*e*. *BRAF*-mutant, EGFR overexpressing colon cancer cell lines), we performed curve shift assays with the EGFR inhibitor gefitinib and the BRAF inhibitors vemurafenib and dabrafenib, in proliferation assays with the *BRAF*-mutant colon carcinoma WiDr cell line (Fig 4). Although we can confirm the poor single agent activities of gefitinib (IC_50_ = 8 μM), vemurafenib as well as dabrafenib show a clear dose-response curve in the WiDr cell proliferation assay, with IC_50_s of 900 nM and 76 nM, respectively (Fig 4). At the highest concentrations, the BRAF inhibitors have only partial efficacy as single agents, whereas in the combinations, when mixed with a relatively small amount of EGFR inhibitor (such as in the dose ratio of 4:1), full efficacy is obtained (Fig 4). Although the mixture curves are not shifted leftward, the CI values indicate synergy, particulary for gefitinib and vemurafenib (Fig 4).

### Cell panel profiling of a drug library

Having demonstrated that our method can identify targeted drug combinations, we proceeded with identifying novel combinations targeted at difficult-to-drug cancer genes. We used a proprietary data set, consisting of the activities of more than hundred small molecule compounds in proliferation assays of forty-four human cancer cell lines, to identify compounds targeting major cancer drivers. The compounds include twenty-eight marketed cytotoxic drugs, all FDA-approved small molecule kinase inhibitors, and many experimental targeted agents, such as modulators of epigenetic targets and experimental kinase inhibitors (Fig 5, S1 Table).

The IC_50_ values of the compounds across cell lines are summarized in Fig 5, where compounds are ranked according to the selective response they display in the cell panel, which is quantified using selectivity entropy (s_sel_). The s_sel_ is a single value summary of the selectivity of each inhibitor, and is high if all cellular IC_50_s are equal (aselective), and low if only one cell line is inhibited potently (selective response) [34]. As expected, the most differentiated profiles, with low entropy values, are obtained from targeted agents, in particular several marketed kinase inhibitors (Fig 5). The ABL inhibitors dasatinib (s_sel_, 0.67) and nilotinib (s_sel_, 0.93), and the growth factor kinase inhibitor cabozantinib (s_sel_, 1.09) are the most selective from all compounds tested (Fig 5). The least differentiated responses, with high entropy values, are from classic cytotoxic agents, such as epothilone B (s_sel_, 3.62), and inhibitors of general cellular processes, such as cell cycle inhibitors and inhibitors of the PI3K pathway (Fig 5). Also remarkable is that a number of cytotoxic agents, such as the DNA synthesis inhibitor cytarabine (s_sel_, 2.84). and the topoisomerase II inhibitor etoposide (s_sel_, 2.83), are comparable to certain targeted inhibitors in terms of cellular selectivity (*cf*. the irreversible EGFR inhibitor pelitinib, s_sel_, 2.82, or the c-Kit inhibitor AB1010 (masitinib), s_sel_, 2.88). Inhibitors of the upcoming target class of epigenetic modulators, such as the bromodomain inhibitors JQ1(s_sel_, 3.60) and I-BET-762 (s_sel_, 3.47), and the histone deacetylase (HDAC) inhibitors entinostat (s_sel_, 3.11) and vorinostat (s_sel,_ 3.50), show only moderate cellular selectivity.

Next, we combined the cellular IC_50_ data with genomic information of the cell line panel to search for drug sensitivity markers. We previously validated this approach with a number of clinically applied kinase inhibitor drugs [31]. Fig 6 demonstrates compounds that specifically inhibit cell proliferation driven by difficult-to-drug cancer genes. For example, ICG-001 was identified by high-throughput screening of a cell line expressing a β-catenin- (*CTNNB1)* dependent reporter gene [39]. Anova analysis of the profiling data from our cell line panel confirms that ICG-001 is more active in inhibiting the proliferation of cell lines that are mutated in *CTNNB1* compared to cell lines that carry the wild-type gene variant (Fig 6A). Another example is the EGFR/ERBB2 inhibitor neratinib, which, like other EGFR inhibitors [31], preferably inhibits the proliferation of cell lines with a mutation in *SMAD4* (Fig 6B). In addition, neratinib preferably inhibits cell lines with amplified *MYC* (Fig 6B). Also GSK-1070916, a potent inhibitor of Aurora B and C kinases, preferably inhibits the proliferation of cell lines with amplified *MYC* (Fig 6C).

### Compounds targeting the *CTNNB1*, *KRAS* or *MYC* oncogenes

Given the high need to treat cancers driven by *CTNNB1*, *KRAS* and *MYC* oncogenes, we extended the Anova analyses of Fig 6 to all compounds tested, and ranked them on the basis of their relative sensitivities for cell lines expressing these cancer genes (Fig 7). We identified eight, five and one compounds with at least a 3.2-fold more potent IC_50_ for *CTNNB1-*mutant, *KRAS-* mutant or *MYC*-amplified cell lines, respectively (Fig 7). These shifts compare favorably with the 3-fold IC_50_ shift observed for the FDA-approved BRAF inhibitor vemurafenib in targeting *BRAF*-mutant cells (Table 1). A t-test was performed to assess if the IC_50_ shifts were significant (p < 0.05, Fig 7). Based on these results, compounds were chosen for combination studies (Table 1). Growth pathway inhibitors (such as MEK or tyrosine kinase inhibitors) were combined with compounds acting through other mechanisms, such as ICG-001, cell cycle inhibitors and taxanes (Fig 7). Preference was given to compounds that showed significant association in the Anova analysis (p < 0.05) or that showed significance in the t-test (p < 0.05) combined with at least 2-fold IC_50_ potentiation (Table 1).

### Targeted combinations for *CTNNB1*-mutant cancers

As compounds that preferentially inhibit *CTNNB1*-mutant cell lines, our profiling identified the MEK inhibitor trametinib and the Wnt-pathway inhibitor ICG-001, both with high significance in the Anova test (Figs 6 and 7, Table 1). Particularly, greater than 10-fold IC_50_ shifts were seen for a series of MEK inhibitors (trametinib, PD-0325901 and AZD-6244) in *CTNNB1*-mutant lines (Fig 7).

Synergistic interactions between trametinib and ICG-001 were investigated in a panel of cell lines, including three *CTNNB1*-wild type colon cancer cell lines, three *CTNNB1*-mutant colon cancer cell lines, and a pair of *CTNNB1-*mutant and *CTNNB1-*wild type lung cancer cell lines (Fig 8 and S3 Fig). Interestingly, and in contrast to our expectations, trametinib and ICG-001 appear to work synergistically in the *CTNNB1*-wild type colon cancer cell line SW620 (Fig 8A), and not in the *CTNNB1-*mutant cell line (Fig 8B). This is confirmed by isobologram analysis (Fig 8C). To generate more reliable CI averages, the experiments were repeated seven times, on several occasions by different scientists (Fig 8, S4 Table). the result is supported by experiments in other cell lines. In the *CTNNB1*-wild type colon cancer cell line HCT 15 synergy is observed, and not in other lines such as HCT 116 (mutant), LoVo (wild type) and LS174T (mutant, S3 Fig). In lung cancer cell lines, the result is more straightforward. Here the combination of ICG-001 and trametinib shows synergy in the *CTNNB1-*mutant cell line (A427, CI_0.5_ = 0.76, SD: 0.11) and not in the wild type cell line (A549, CI_0.5_ = 1.03, SD: 0.06, S3 Fig).

### Targeted combinations for *MYC*-amplified cancer

In the cell line panel, the EGFR/ERBB2 inhibitor neratinib, the Aurora inhibitor GSK-1070916, the PLK1 inhibitor BI-2536 and the taxane paclitaxel preferentially inhibit *MYC*-amplified cell lines (Fig 7, Table 1). The p-values from the t-test and the presence of inhibitors with similar mechanisms in the list of *MYC*-targeted compounds indicate that the effects are real (Fig 7). For instance, other Aurora inhibitors (MLN-8054 and AMG-900), another taxane (docetaxel), another PLK1 inhibitor (GSK-461364) and other EGFR/ERBB2 inhibitors (afatinib, lapatinib) are also among the *MYC*-targeted compounds (Fig 7).

We tested combinations of the EGFR/ERBB2 inhibitor neratinib with the cell cycle inhibitors (BI-2536, GSK-1070916 and paclitaxel or docetaxel) in the *MYC*-amplified cancer cell line NCI-H82. All combinations showed synergy. Neratinib with the Aurora inhibitor GSK-1070916 and neratinib with a taxane were further tested in three sets of cell lines with amplified or non-amplified *MYC*, from breast, colon and lung cancers (Figs 9, 10 and S4, S5 Figs).

Neratinib and GSK-1070916 show synergy in the *MYC*-amplified breast cancer line AU-565, but not in the wild-type control BT549 (Fig 9A and 9B). Therefore, in this context, neratinib and GSK-1070916 are a targeted combination. In colon and lung cancer cell lines, the combination shows general synergistic effects, and higher efficacy in *MYC*-amplified lung cancer cell lines (S4 Fig).

Neratinib and paclitaxel show synergy in the *MYC*-amplified lung cancer line NCI-H82, *i*.*e*. the combination induces a complete response, while the single agents show only a partial effect (Fig 10A). This is not the case in the non-amplified control A427 (Fig 10B). Combination of neratinib with another taxane, docetaxel, confirms this synergy in the same pair of cell lines, (S5 Fig). In colon cancer cell lines, there is a more general synergistic effect. In breast cancer cell lines, neratinib and docetaxel show a synergistic effect specific for the *MYC*-amplified line (S5 Fig). This indicates that the combination of neratinib and a taxane can be targeted, depending on tumor cell type.

Interestingly, the observed synergies for neratinib and taxanes are similar to those of the neratinib and GSK-1070916 combination (compare for instance Fig 9 with S5D and S5E Fig, or Fig 10 with S4C and S4D Fig).

### Targeted combinations for *KRAS*-mutant cancer

The cell line profiling revealed that, among others, the ERBB2 kinase inhibitor TAK-165, the Aurora kinase inhibitor MLN-8054 and the MEK kinase inhibitor trametinib specifically inhibit proliferation of *KRAS*-mutant cell lines (Fig 7, Table 1). These findings are confirmed by the presence of other MEK inhibitors (PD-0325901 and AZD-6244) and another Aurora inhibitor (AMG-900) in the list of *KRAS*-mutant-targeted compounds (Fig 7). The activity of TAK-165 is not confirmed by other EGFR or ERBB2 inhibitors.

To search for targeted synergies, we combined the growth factor signaling inhibitor TAK-165 with an inhibitor of proliferation (trametinib) or a cell cycle inhibitor (MLN-8054) in proliferation assays with the *KRAS*-mutant A427 lung cancer cell line (Fig 11, S7 and S8 Figs). Particularly the combination of TAK-165 and trametinib shows synergy (Fig 11). This combination was further profiled in a *KRAS*-wild type lung cancer cell line (Fig 11B), and a pair of *KRAS*-mutant and wild type colon cancer cell lines (S7 Fig). Synergy appears in both lung cancer cell lines, but in none of the colon cancer cell lines (S7 Fig), indicating that the synergy of TAK-165 and trametinib depends on tissue type.

## Discussion

### Targeted synergy

Combination treatment is a cornerstone of cancer chemotherapy and there is great interest to identify new combinations *in vitro* to improve treatment outcome [1–6]. However, truly synergistic combinations are rare [4]. Here, we describe a novel work flow to study compound synergy. We applied this approach to validate a number of literature examples, and subsequently, used it to identify novel combinations of existing drugs that specifically target difficult-to-drug cancer genes. First, we compared curve shift analysis with combination matrix screening. We found that curve shift analysis is a more robust method than combination matrix screening with Bliss-scoring. The signal-to-noise ratios of synergistic and additive control experiments (Figs 1D, 2C and 2D) show that curve shift analysis has the least inter-assay variation. However, we want to point out that the CI_0.5_ only takes one response level (50%) into account and that it remains important to visually inspect curve shape. The superiority of the curve shift method, compared to Bliss methods, most likely arises from its analysis of IC_50_s, rather than maximum effects, which are more prone to variation.

The curve shift method confirms the synergistic activity of MEK inhibitors, such as trametinib, with PI3K inhibitors, such as GDC-0941 (Fig 1), and the synergistic activity of trametinib with the BRAF inhibitors dabrafenib and vemurafinib (Fig 3) [28]. Curve shift and CI analysis shows that trametinib is more synergistic with dabrafenib than with vemurafinib. This correlates with the higher selectivity of dabrafenib for *BRAF*-mutant cell lines in comparison to vemurafenib, which in turn is related to the higher biochemical potency of dabrafenib [31].

We also confirm the synergistic activity of BRAF and EGFR inhibitors in *BRAF*-mutant colon cancer cell lines (Fig 4) [29, 30].The claim that EGFR and BRAF inhibitors are inactive in *BRAF*-mutant colon cancer lines appears unfounded however [29, 30], as we observed a clear IC_50_ response (Fig 4). Interestingly, there is an increase in maximum efficacy of dabrafenib and vemurafenib induced by low concentrations of EGFR inhibitors (Fig 4). In combination with gefitinib, vemurafenib seems to be more synergistic than dabrafenib, which might be related to the better biochemical selectivity of vemurafenib [31]. The superiority of vemurafenib here contrasts with the superiority of dabrafenib in combination with trametinib (Fig 3); it seems that both BRAF-dependent biological systems have their own optimal set of synergistic inhibitors.

We also used our setup to study if synergistic interactions are exclusive to (a specific subset of) cancer cells, and whether synergistic toxicities are absent in non-transformed cells, or in cells carrying a wild-type variant of the oncogene. The combination of the PI3K and MEK inhibitor not only shows synergy in cancer cells but also in non-transformed fibroblasts, a model for normal, non-malignant cells (Fig 1E). This is in line with the clinical observation that the combination has significant additional toxicity [40].

The combination of dabrafenib and trametinib is more specific in its synergistic interaction, as it only occurs in *BRAF*-mutant cell lines, not in *BRAF*-wild type cell lines (Fig 3). In fibroblasts the combination is antagonistic (S2 Fig). This is in line with the limited additional toxicity observed in combination treatment in the clinic [41]. This, and the subsequent FDA approval for dabrafenib with trametinib, supports the notion that synergistic combinations are interesting for follow-up, but that *targeted* synergistic combinations such as trametinib and dabrafenib are even more promising for further *in vivo* evaluation.

The example of dabrafenib and trametinib moreover shows that compounds that target the same subset of cancer cells (*i*.*e*., *BRAF*-mutant) can act synergistically. We built on this notion in our selection of drug combinations to target *CTNNB1*, *KRAS* and *MYC-*driven cell growth.

### Targeting *CTNNB1*-mutations

Mutations in the *CTNNB1* (β-catenin) gene affect the Wnt pathway and occur in a high percentage of uterine and colorectal cancers, and medullo-blastomas [42,43]. We identified two inhibitors that potently and significantly inhibit cells expressing mutant *CTNNB1*: trametinib and ICG-001 (Fig 6, Table 1). This suggests that these compounds might find an application for treatment of this cancer subtype. The targeting effect of trametinib is consistent with its role in the synthesis of β-catenin [44]; that of ICG-001 is consistent with its known inhibition of signaling downstream of β-catenin in the Wnt-pathway [39].

Subsequent combination studies revealed synergic interaction between trametinib and ICG-001 (Fig 8) in the *CTNNB1*-wild type SW620 colon cancer cell line and not in the *CTNNB1*-mutant colon cancer line SW48 (Fig 8). Although this seems counterintuitive, the SW620 line contains an *APC* mutation, and thereby is also dependent on Wnt signalling for growth (S2 Table). In fact, all colon cancer cell lines tested contain Wnt signalling mutations, though only in two, synergy is observed (S3 Fig). These (HCT-115 and SW620) are the only ones that have *TP53* mutations in addition to Wnt signalling mutations (S2 Table). It is known that wild type *TP53* can contribute to drug resistance in colon cancer cell lines [45] and cells with wild type TP53 were relatively resistant to trametinib in a profiling study in a 44-cell line panel [31]. Thus, in colon cancer, ICG-001 and trametinib might only show synergy in presence of dual Wnt-pathway and *TP53* mutations. In lung cancer cell lines, trametinib and ICG-001 show synergy in the cell line with a Wnt-pathway (*CTNNB1)* mutation and not in the wild type control (S3 Fig). Therefore, trametinib and ICG-001 are a synergistic drug pair in particular subsets of cell lines.

### Targeting *MYC*-amplifications

Amplification of *MYC* occurs in many cancers, including more than 40% of ovarian cancers [42,43]. Despite being one of the most investigated oncogenes, there are currently no therapies that target MYC signaling specifically [21, 22]. We discovered that a number of Aurora and PLK1 kinase inhibitors were relatively selective for *MYC*-amplified cells, as well as paclitaxel, docetaxel and neratinib (Table 1). Since GSK-1070916 and BI-2536 belong to the most selective kinase inhibitors developed so far [46], it appears that indeed Aurora B/C and PLK1 inhibition are responsible for the selective targeting of *MYC*-amplified cells. These results agree with RNAi-based synthetic lethality studies, which have identified Aurora B inhibition as synthetically lethal with *MYC*-driven cell growth [20, 47].

Neratinib is a spectrum-selective EGFR/ERBB2 inhibitor [48]. The presence of other highly selective EGFR/ERBB2 inhibitors such as afatinib and lapatinib among the list of *MYC*-targeting agents (Fig 7), and the absence of selective EGFR inhibitors, such as gefitinib and erlotinib, suggests that ERBB2 inhibition, or inhibition of both EGFR and ERBB2 is responsible for *MYC*-targeting. This is supported by recent *in vitro* and *in vivo* studies that show that *ERBB2* and *MYC* can cooperate to induce aggressiveness in breast cancer [49].

Subsequent combination studies showed synergistic interaction between neratinib and GSK-1070916 in a variety of cancer cell lines. The results in breast cancer lines (Fig 9) show that neratinib and GSK-1070916 can be a targeted combination in the context of *MYC* amplification. Other groups have also found synergy between EGFR/ERBB2 and Aurora inhibition, either in genomic or pharmacological studies [50–53]. However, the synergy in context of *MYC*-amplification has not been described before, to our knowledge. Interestingly, the AU-565 cell line that shows the best synergy (Fig 9A) also has an *ERBB2* amplification in addition to *MYC* (S2 Table). It is known that MYC is a downstream effector of ERBB2 signaling [54], that *MYC*-amplification results in Aurora kinase- dependent growth [47], and that co-amplification of *ERBB2* and *MYC* is a frequent event in cancer [55]. Therefore, we propose that *ERBB2*, *MYC* and Aurora form an integrated oncogenic signaling network that is optimally inhibited by the combination of neratinib and GSK-1070916 in *MYC*-amplified cells.

A second combination that shows promise is that of neratinib and taxanes such as paclitaxel and docetaxel. This combination also shows MYC-dependent synergistic interaction in lung cancer and breast cancer cell lines (Fig 10 and S5 Fig), similar to the combination of neratinib and GSK-1070916. This might be because both taxanes and the Aurora inhibitor GSK-1070916 impact cell cycle progression. In general, the synergistic interactions are less strong for the taxane combinations. The combination of neratinib and paclitaxel is currently tested in the clinic for treatment of breast cancer [56]. Our data support further experiments with this combination in other indications.

### Targeting *KRAS-*mutations

KRAS activation occurs in more than 70% of pancreatic cancers and the identification of specific inhibitors or combinations that inhibit KRAS signaling has proven to be difficult [57]. Our screen identified three types of kinase inhibitors that target *KRAS*-driven cell growth: various MEK inhibitors, including trametinib, the ERBB2 inhibitor TAK-165, and Aurora kinase inhibitors, such as MLN-8054 and AMG-900 (Fig 7). MEK is part of the RAS pathway and it has been shown before that *RAS*-mutant cell lines are sensitive to MEK inhibitors [36]. Aurora kinases have never been implicated in targeting of *KRAS*-mutations, despite a wealth of synthetic lethal studies [20, 21, 57, 58]. MLN-8054 is a selective Aurora A inhibitor [48], AMG-900 has pan-Aurora activity [48]. This, and the absence of the selective Aurora B/C inhibitor GSK-1070916 in the list, suggests that the selective *KRAS*-targeting is due to inhibition of Aurora A. This is further supported by data on the Aurora A/B inhibitor ZM-447439, which shows significantly more potent IC_50_s on *KRAS*-mutant cell lines in a 1000-cell line screen of the Sanger centre [9].

TAK-165 [59] is the only growth factor signaling inhibitor in our screen that shows *KRAS*-targeting. Interestingly, it is also the most *KRAS*-targeted compound (Fig 7). Recently, it was described that ERBB2 and ERBB3 signaling play an important role in *KRAS*-dependent cell growth [29, 60]. It is, however, unclear why TAK-165 is so particularly adept at targeting *KRAS*, while other inhibitors that inhibit ERBB2, such neratinib, lapatinib or afatinib, are not. TAK-165 distinguishes itself from these other inhibitors in that it does not inhibit EGFR [46, 59]. We conclude that inhibition of ERBB2 without simultaneous inhibition of EGFR might contribute to targeting *KRAS*-mutant cell lines.

Combination studies reveal trametinib and TAK-165 as a synergistic pair particularly in lung cancer cell lines (Fig 11). The biological significance of this combination is supported by the recent results of Sun *et al*., who showed that a MEK and ERBB2 inhibitor act synergistically in *KRAS*-mutant cell lines [60]. In comparison with this work, our study explored a broader range of inhibitors, and we identified TAK-165 and trametinib as the best pair to take forward. Because both compounds have been already tested in humans, they provide excellent candidates for validating the concept of dual inhibition of MEK and ERBB2 in more advanced models of *KRAS*-mutant cancer.

## Conclusion

We have developed a workflow to identify synergistic combinations based on combining compounds that target the same cancer driver genes or pathways. In agreement with earlier, large scale combination studies, our experience is that truly synergistic combinations are rare. For instance, Borisy *et al*. who screened 100,000 combinations identified forty-one synergistic combinations (0.04%) [4]. Cokol *et al*. who screened two hundred combinations identified thirty-eight (19%) [5]. Using our rational approach, that combines compounds that target similar cancer cell line subsets, we discovered six novel synergistic combinations, of eight tested, a success rate of 75%. Despite the limited experience with this approach, it seems that compounds that are cellularly more selective also give rise to more potent synergies. A good example is dabrafenib, which is more cellularly selective for cells that grow dependent on BRAF compared to vemurafenib [31], and is also the superior partner of the MEK inhibitor trametinib. For PI3K inhibitors in combination with MEK inhibitors, we have also observed that the most cellularly selective inhibitors result in the most potent synergy. However, there are also exceptions, such as our study of EGFR and BRAF-inhibitors shows, in which vemurafenib is superior to dabrafenib. The new compound combinations that we have discovered are worth further study. All compounds in the suggested combinations have already been in clinical trials—except ICG-001- and therefore can rapidly progress to further *in vivo* validation. The generation of increasingly sophisticated targeted combinations, assembled on the basis of *in vitro* data, will be an important step in overcoming efficacy and resistance problems associated with targeted single agent therapy.

## Supporting Information

S1 Table Sources of inhibitors used in the cell line profiling (XLSX)

S2 Table Overview of the cell lines in the Oncolines cell panel and presence of twenty-three commonly occurring cancer genes (XLSX)

S3 Table IC_50_s of compounds studied in synergy experiments in forty-four cell line Oncolines panel (XLSX)

S4 Table CI values of all compound combinations tested (XLSX)

S1 Equation Formula for Bliss parameterfitting (PDF)

S1 Fig Curve shift experiment of the combination of GDC-0941 and AZD-6244 in the hTERT immortalized cell line BJ5-ta (PDF)

S2 Fig Curve shift experiment of the combination of trametinib and dabrafenib in the hTERT immortalized cell lines BJ5-ta and RPE-1 (PDF)

S3 Fig Curve shift experiments of the combination of ICG-001 and trametinib (PDF)

S4 Fig Curve shift experiments of the combination of neratinib (HKI-272) and GSK-1070916 (PDF)

S5 Fig Curve shift experiments of the combination of neratinib (HKI-272) and docetaxel (PDF)

S6 Fig Curve shift experiments of the combination of neratinib (HKI-272) and BI-2536 (PDF)

S7 Fig Curve shift experiments of the combination of trametinib and TAK-165 (PDF)

S8 Fig Curve shift experiments of the combination of MLN-8054 and TAK-165 (PDF)
