# Supplementary material for: Selective Targeting of CTNNB1-, KRAS- or MYC-Driven Cell Growth by Combinations of Existing Drugs
Source: PLoS One. 2015 May 27;10(5):e0125021. doi: 10.1371/journal.pone.0125021 (PMC4446296; doi:10.1371/journal.pone.0125021)
Supplement: S1 Equation — (PDF) [file pone.0125021.s005.pdf]

$$minimization\ target = \sum_{ab}(f_{ab} - \beta \cdot f_a f_b)^2 \qquad \text{(S1)}$$

Supplementary Equation S1. Formula for Bliss parameter fitting. After ref. [5]. For an experiment as indicated in Figure 2, with rows labelled *a* and columns *b*. For a well, the fractional growth effect measured is f_{ab}. The fractional growth effects of the single agents at similar concentrations are f_a and f_b. The total squared difference is a variant of the Bliss score [4], with an additional parameter β. The function (S1) was minimized using the solver function in Microsoft Excel. If β > 0, the experiment shows synergy. If β < 0, there is antagonism.
