# Supplementary material for: Selective Targeting of CTNNB1-, KRAS- or MYC-Driven Cell Growth by Combinations of Existing Drugs
Source: PLoS One. 2015 May 27;10(5):e0125021. doi: 10.1371/journal.pone.0125021 (PMC4446296; doi:10.1371/journal.pone.0125021)

Supplementary Figure S1. Curve shift experiments of the combination of the MEK inhibitor AZD-6244 (green) and the PI3K inhibitor GDC-0941 (blue) in the BJ-5ta human foreskin fibroblast cell line, immortalized using hTERT. In this assay, AZD-6244 and GDC-0941 show synergy. Mixture ratios used were 1:1, red; 4:1, orange; 1:4, yellow. CI values and standard deviations (SD) are based on three mixtures (see Table S4 for individual values).

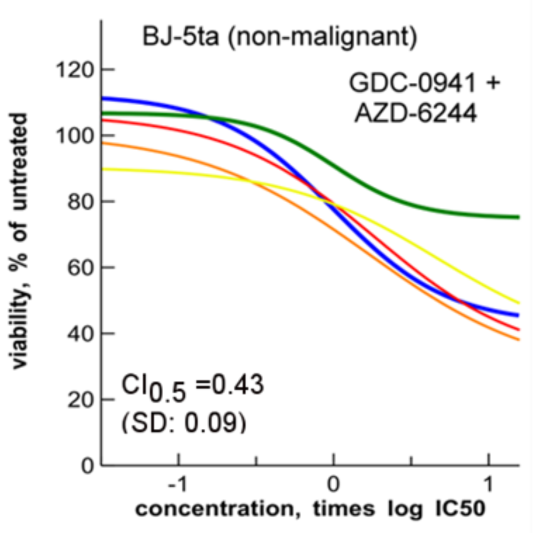

Supplement: S1 Fig — (PDF) [file pone.0125021.s006.pdf]
