# Supplementary material for: Selective Targeting of CTNNB1-, KRAS- or MYC-Driven Cell Growth by Combinations of Existing Drugs
Source: PLoS One. 2015 May 27;10(5):e0125021. doi: 10.1371/journal.pone.0125021 (PMC4446296; doi:10.1371/journal.pone.0125021)

Supplementary Figure S2. Curve shift experiments of the combination of trametinib (green) and dabrafenib (blue) in the BJ-5ta human foreskin fibroblast cell line, and the RPE-1 retinal pigment epithelial cell line, both immortalized with hTERT. In both assays, dabrafenib and trametinib act antagonistically. Mixture ratios used were 1:1, red; 4:1, orange; 1:4, yellow. CI values and standard deviations (SD) are based on three mixtures (see Table S4 for individual values).

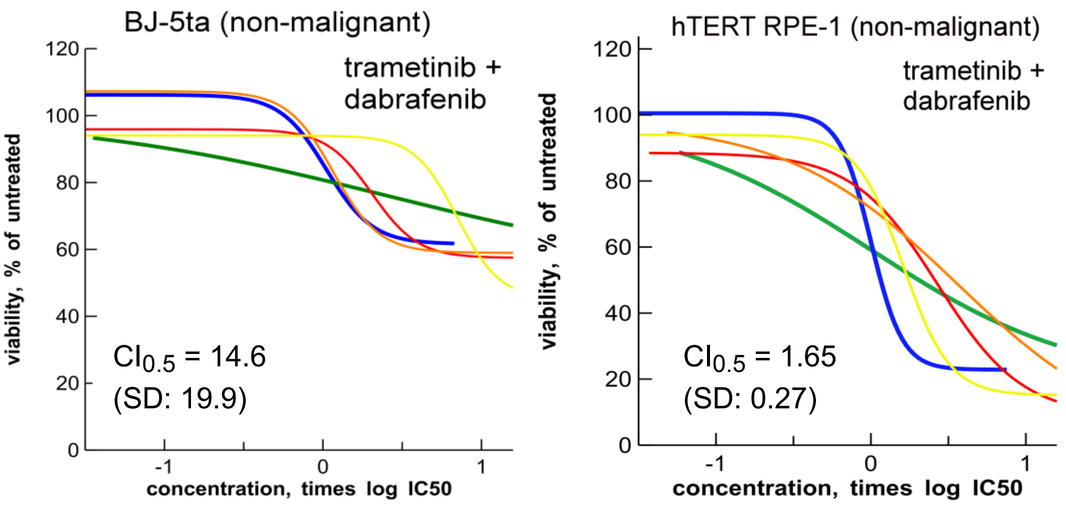

Supplement: S2 Fig — (PDF) [file pone.0125021.s007.pdf]
