# Supplementary material for: Selective Targeting of CTNNB1-, KRAS- or MYC-Driven Cell Growth by Combinations of Existing Drugs
Source: PLoS One. 2015 May 27;10(5):e0125021. doi: 10.1371/journal.pone.0125021 (PMC4446296; doi:10.1371/journal.pone.0125021)
Supplement: S3 Fig — (PDF) [file pone.0125021.s008.pdf]

Supplementary Figure S3. Curve shift experiments of the combination of ICG-001 (green) and trametinib (blue) in various cell lines. Mixture ratios used were 1:1, red; 4:1, orange; 1:4, yellow. CI values and standard deviations (SD) are based on three mixtures (see Table S4 for individual values)

CTNNB1-mutant colon cancer cell lines

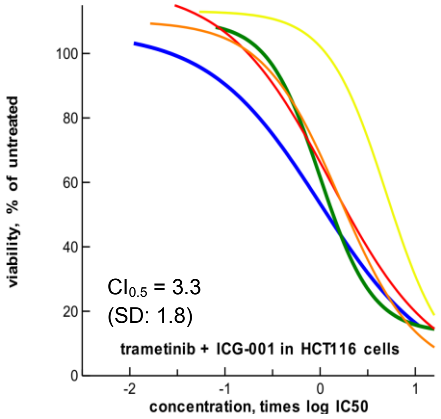

A

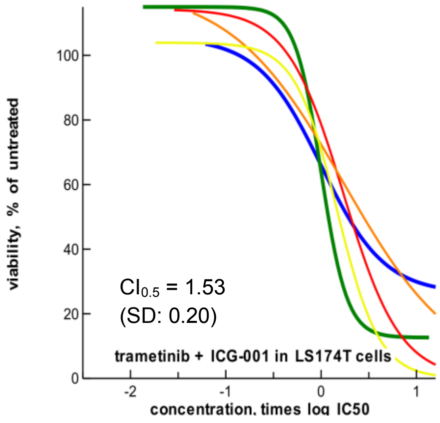

B

CTNNB1-wild type colon cancer cell lines

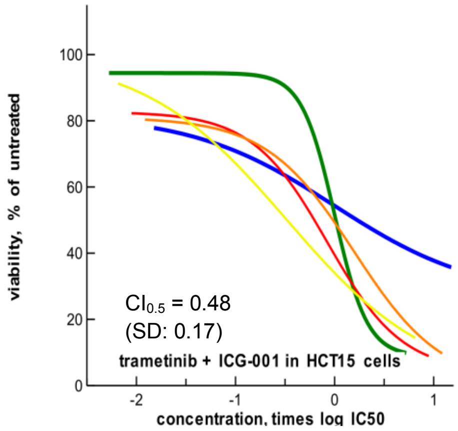

C

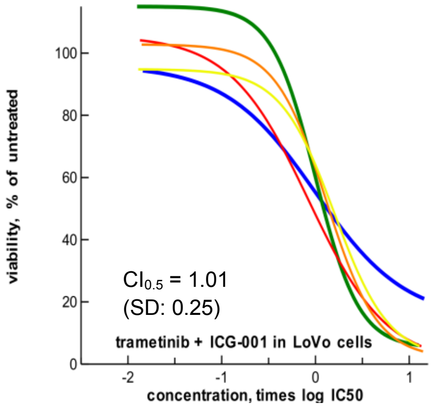

D

CTNNB1-mutant lung cancer cell lines

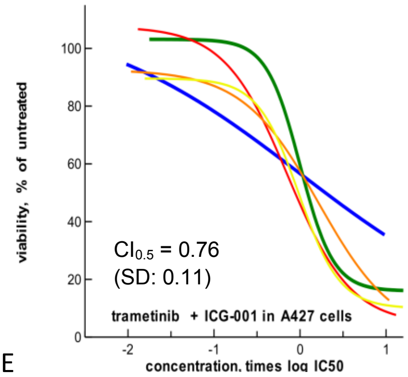

E

CTNNB1-wild type lung cancer cell lines

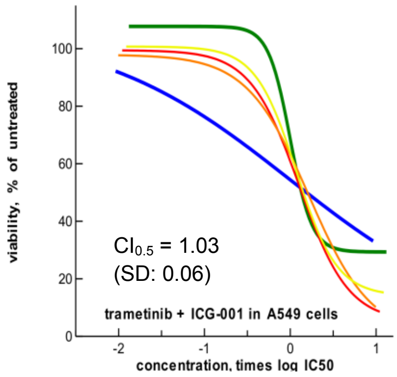

F
