# Supplementary material for: Selective Targeting of CTNNB1-, KRAS- or MYC-Driven Cell Growth by Combinations of Existing Drugs
Source: PLoS One. 2015 May 27;10(5):e0125021. doi: 10.1371/journal.pone.0125021 (PMC4446296; doi:10.1371/journal.pone.0125021)

Supplementary Figure S4. Curve shift experiments of the combination of neratinib (HKI-272, blue) and GSK-1070916 (green) in various cell lines. For panels **A** and **B**, the 100 % effect level was set to the response at the highest concentration tested. Mixture ratios used were 1:1, red; 4:1, orange; 1:4, yellow. CI values and standard deviations (SD) are based on three mixtures (see Table S4 for individual values)

Colon cancer cell lines. *MYC*-amplified (left) and non-*MYC*- amplified (right)

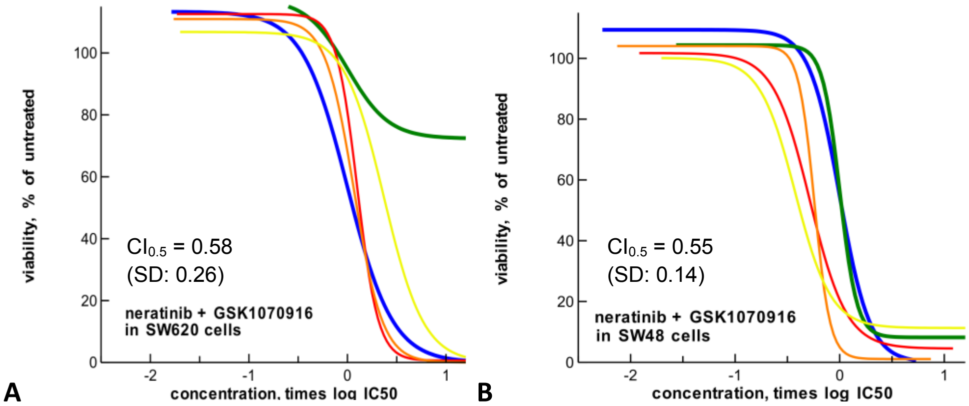

Lung cancer cell lines. *MYC*-amplified (left) and non-*MYC* -amplified (right)

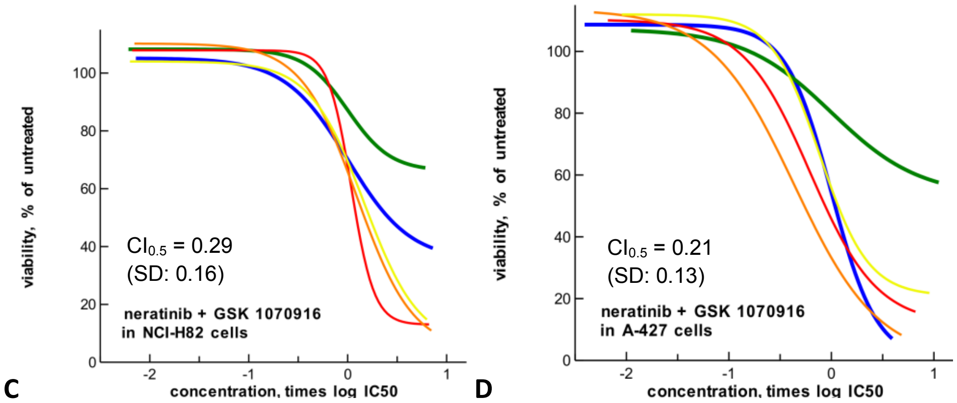

Supplement: S4 Fig — (PDF) [file pone.0125021.s009.pdf]
