# Supplementary material for: Selective Targeting of CTNNB1-, KRAS- or MYC-Driven Cell Growth by Combinations of Existing Drugs
Source: PLoS One. 2015 May 27;10(5):e0125021. doi: 10.1371/journal.pone.0125021 (PMC4446296; doi:10.1371/journal.pone.0125021)
Supplement: S5 Fig — (PDF) [file pone.0125021.s010.pdf]

Supplementary Figure S5. Curve shift experiments of the combination of neratinib (HKI-272, blue) and docetaxel (green) in various cell lines. For panel **G**, the 100 % effect level was set to the response at the highest concentration tested. In panel **C**, one ratio was omitted because of technical failure. Mixture ratios used were 1:1, red; 4:1, orange; 1:4, yellow. CI values and standard deviations (SD) are based on three mixtures (see Table S4 for individual values)

Lung cancer cell lines. *MYC*-amplified (left) and non-*MYC*- amplified (2 panels on the right)

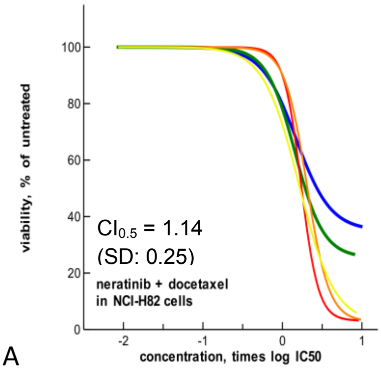

A

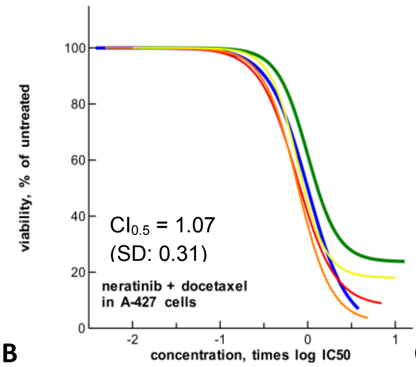

B

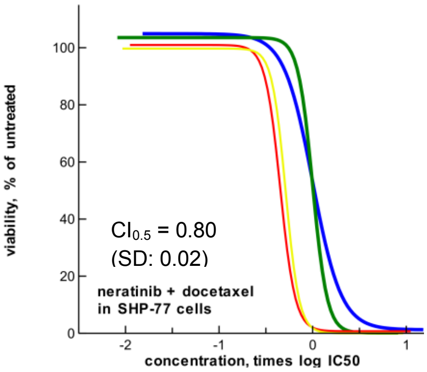

C

Breast cancer cell lines. *MYC*-amplified (left) and non-*MYC*- amplified (right)

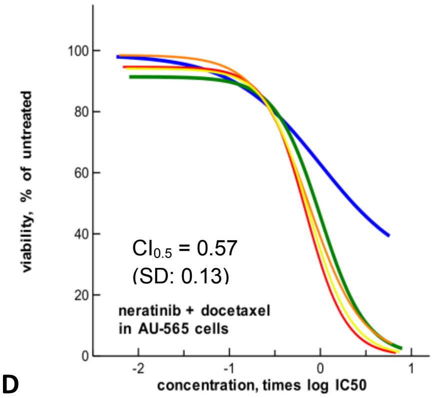

D

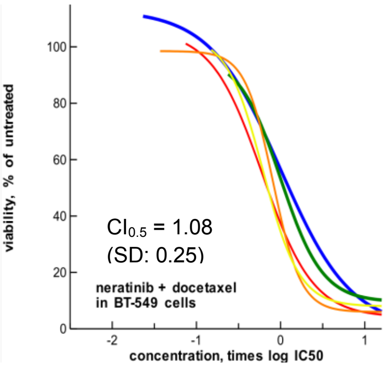

E

Colon cancer cell lines. *MYC*-amplified (left) and non-*MYC*- amplified (right)

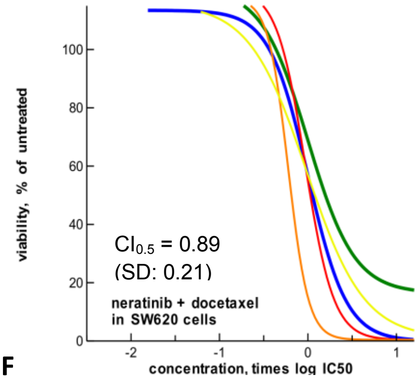

F

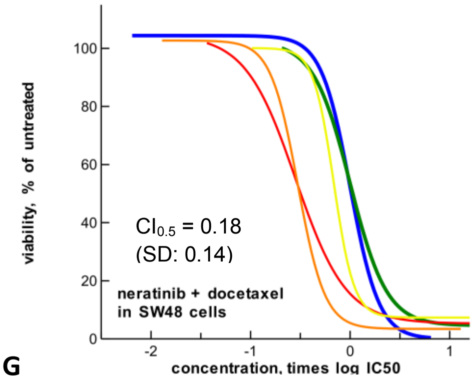

G
