# Supplementary material for: Selective Targeting of CTNNB1-, KRAS- or MYC-Driven Cell Growth by Combinations of Existing Drugs
Source: PLoS One. 2015 May 27;10(5):e0125021. doi: 10.1371/journal.pone.0125021 (PMC4446296; doi:10.1371/journal.pone.0125021)
Supplement: S7 Fig — (PDF) [file pone.0125021.s012.pdf]

Supplementary Figure S7. Curve shift experiments of the combination of trametinib (green) and TAK-165 (blue) in colon cancer cell lines. Mixture ratios used were 1:1, red; 4:1, orange; 1:4, yellow. CI values and standard deviations (SD) are based on three mixtures (see Table S4 for individual values)

Colon cancer cell lines. *KRAS*-mutated (left) and non-*KRAS*mutated (right)

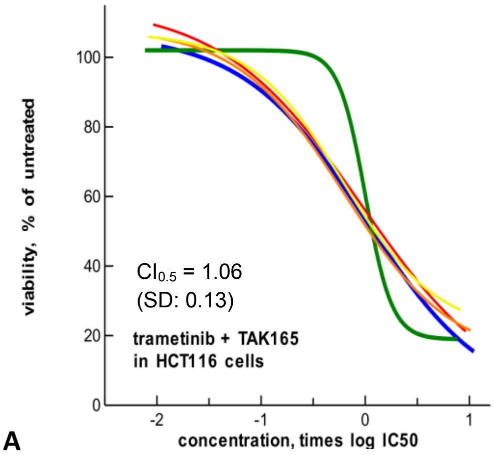

A

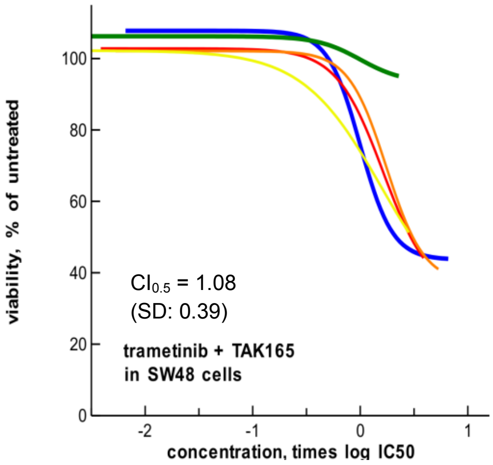

B
