# Supplementary material for: Selective Targeting of CTNNB1-, KRAS- or MYC-Driven Cell Growth by Combinations of Existing Drugs
Source: PLoS One. 2015 May 27;10(5):e0125021. doi: 10.1371/journal.pone.0125021 (PMC4446296; doi:10.1371/journal.pone.0125021)

Supplementary Figure S8. Curve shift experiment of the combination of MLN-8054 (green) and TAK-165 (blue).Mixture ratios used were 1:1, red; 4:1, orange; 1:4, yellow. CI values and standard deviations (SD) are based on three mixtures (see Table S4 for individual values)

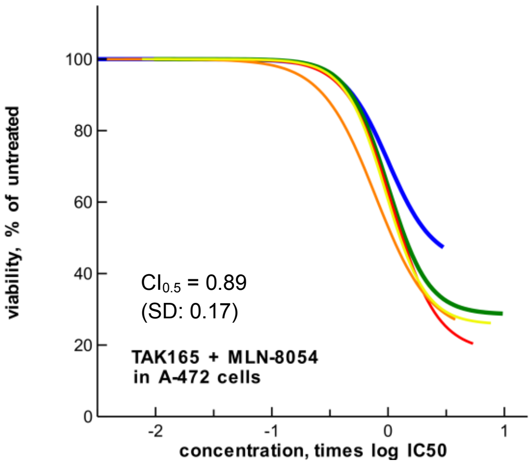

Supplement: S8 Fig — (PDF) [file pone.0125021.s013.pdf]
