# Supplementary material for: Correction: Selective Targeting of CTNNB1-, KRAS- or MYC-Driven Cell Growth by Combinations of Existing Drugs
Source: PLoS One. 2015 Jun 29;10(6):e0132230. doi: 10.1371/journal.pone.0132230 (PMC4488309; doi:10.1371/journal.pone.0132230)
Supplement: S2 File — (PDF) [file pone.0132230.s002.pdf]

RESEARCH ARTICLE

Selective Targeting of *CTNNB1*-, *KRAS*- or *MYC*-Driven Cell Growth by Combinations of Existing Drugs

Joost C. M. Uitdehaag, Jeroen A. D. M. de Roos, Antoon M. van Doornmalen, Martine B. W. Prinsen, Jill A. P. Spijkers-Hagelstein, Judith R. F. de Vetter, Jos de Man, Rogier C. Buijsman, Guido J. R. Zaman*

Netherlands Translational Research Center B.V., Oss, The Netherlands

* guido.zaman@ntrc.nl

Abstract

The aim of combination drug treatment in cancer therapy is to improve response rate and to decrease the probability of the development of drug resistance. Preferably, drug combinations are synergistic rather than additive, and, ideally, drug combinations work synergistically only in cancer cells and not in non-malignant cells. We have developed a workflow to identify such targeted synergies, and applied this approach to selectively inhibit the proliferation of cell lines with mutations in genes that are difficult to modulate with small molecules. The approach is based on curve shift analysis, which we demonstrate is a more robust method of determining synergy than combination matrix screening with Bliss-scoring. We show that the MEK inhibitor trametinib is more synergistic in combination with the BRAF inhibitor dabrafenib than with vemurafenib, another BRAF inhibitor. In addition, we show that the combination of MEK and BRAF inhibitors is synergistic in *BRAF*-mutant melanoma cells, and additive or antagonistic in, respectively, *BRAF*-wild type melanoma cells and non-malignant fibroblasts. This combination exemplifies that synergistic action of drugs can depend on cancer genotype. Next, we used curve shift analysis to identify new drug combinations that specifically inhibit cancer cell proliferation driven by difficult-to-drug cancer genes. Combination studies were performed with compounds that as single agents showed preference for inhibition of cancer cells with mutations in either the *CTNNB1* gene (coding for β-catenin), *KRAS*, or cancer cells expressing increased copy numbers of *MYC*. We demonstrate that the Wnt-pathway inhibitor ICG-001 and trametinib acted synergistically in Wnt-pathway-mutant cell lines. The ERBB2 inhibitor TAK-165 was synergistic with trametinib in *KRAS*-mutant cell lines. The EGFR/ERBB2 inhibitor neratinib acted synergistically with the spindle poison docetaxel and with the Aurora kinase inhibitor GSK-1070916 in cell lines with *MYC* amplification. Our approach can therefore efficiently discover novel drug combinations that selectively target cancer genes.

OPEN ACCESS

Citation: Uitdehaag JCM, de Roos JADM, van Doornmalen AM, Prinsen MBW, Spijkers-Hagelstein JAP, de Vetter JRF, et al. (2015) Selective Targeting of *CTNNB1*-, *KRAS*- or *MYC*-Driven Cell Growth by Combinations of Existing Drugs. PLoS ONE 10(5): e0125021. doi:10.1371/journal.pone.0125021

Academic Editor: Nikolas K. Haass, University of Queensland Diamantina Institute, AUSTRALIA

Received: December 7, 2014

Accepted: March 19, 2015

Published: May 27, 2015

Copyright: © 2015 Uitdehaag et al. This is an open access article distributed under the terms of the Creative Commons Attribution License, which permits unrestricted use, distribution, and reproduction in any medium, provided the original author and source are credited.

Data Availability Statement: All relevant data are within the paper and its Supporting Information files.

Funding: This research was supported by a grant from the Innovation Office of the Ministry of Economic Affairs of the Netherlands (INT 111039). Netherlands Translational Research Center B. V. provided support in the form of salaries for authors JCNU, JADMR, AMVD, MBWP, JAPSH, JDM, RCB and GJRZ, but did not have any additional role in study design, data collection and analysis, decision to publish, or preparation of the manuscript. The specific roles of

these authors are articulated in the 'author contributions' section.

Competing Interests: RCB and GJRZ are founders and shareholders of Netherlands Translational Research Center B. V. (NTRC). The described cancer cell line profiling and combination testing is offered as a commercial service by NTRC under the brand name SynergyFinder. This does not alter the authors' adherence to PLOS ONE policies on sharing data and materials.

Introduction

The aim of combination drug treatment in cancer therapy is to achieve improved response rates and to decrease the probability of the development of drug resistance [1–3]. The discovery of new effective drug combinations is, however, constrained by the costs of carrying out systematic combination studies in the clinic and by the large number of possible drug combinations [4–6]. Cancer cell lines are an attractive model to investigate new drug combinations because they can be used to determine whether new combinations are truly synergistic, as opposed to additive [7, 8]. Moreover, cancer cell lines provide a good representation of the diversity of genetic changes that drive human cancers [9, 10].

In the past three decades the molecular causes of most of the major cancers have been identified, and this has led to the development of a number of medicines that target specific signaling pathways that are perturbed in cancer. Examples are imatinib, targeting a specific fusion protein of ABL kinase in chronic myeloid leukemia [11], and vemurafenib and dabrafenib, targeting a mutant form of the protein kinase BRAF in metastatic melanoma [12, 13]. These targeted therapies bring great benefit to patients, because they improve survival rates with less side effects than traditional, less selective, cytotoxic drugs. However, available targeted therapies are only beneficial to a small fraction of cancer patients, while after an initial good response, drug resistance often develops, similar to treatment with cytotoxic agents [14]. Furthermore, for some of the most frequently occurring oncogenic drivers, such as β-catenin (encoded by the gene *CTNNB1)*, KRAS and MYC, it has been shown to be difficult to identify small molecules that modulate these targets specifically, and these targets are therefore referred to as 'undruggable' [15].

Targeting of cell growth driven by undruggable cancer genes has been shown by RNA interference (RNAi)-based screenings. Knock-down of downstream, parallel, or compensatory mechanisms can lead to the selective killing of cells that express mutant *CTNNB1*, *KRAS*, or overexpress *MYC* [16–24]. However, attempts to translate these 'synthetic-lethal' studies to drug therapy have largely failed due to lack of efficacy (compare, *e.g.*, references [19] and [25]). Potentially, better responses could be obtained by inhibiting multiple pathways downstream of the cancer gene, which is often a central signaling node. Such an approach of dual inhibition has never been tested, primarily because it is difficult to make a dose-range of inhibition of target activity with RNAi, and to apply a knock-down on two targets simultaneously. Pharmacological interference has the potential to resolve these problems, because it is feasible to target multiple pathways, and because results can be directly translated to relevant in *vivo* efficacy models [26].

There are some exciting examples of synergistic drug combinations involving targeted inhibitors. For instance, Liu *et al.* showed that inhibition of the MAPK pathway with MEK inhibitors acts synergistically with inhibition of the PI3K pathway [27]. Investigators from GlaxoSmithKline showed that the MEK inhibitor trametinib acts synergistically with the BRAF inhibitor dabrafenib, although both inhibitors act in the same pathway [28]. The combination of the two drugs is approved by the U.S. Food and Drug Administration (FDA) for the treatment of *BRAF*-mutant metastatic melanoma. A third combination of targeted inhibitors are BRAF and EGFR inhibitors in *BRAF*-mutant colorectal cancer. The BRAF inhibitor vemurafenib lacks efficacy in this cancer, due to a feedback regulatory mechanism resulting in high EGFR activity [29,30]. This feedback mechanism can be suppressed by combination with EGFR inhibitors [29,30]. All three examples show that synergistic effects between targeted drugs are possible, whether they inhibit different, or similar pathways.

It is important to consider that the signaling pathways that are inhibited by targeted drugs, such as the PI3K, MEK, BRAF and EGFR pathways, are also essential for proliferation of

normal, non-transformed cells. For this reason, many targeted inhibitors are also toxic to cells that do not express specific oncogenes. If these toxicities would also synergize, the combination of two compounds would not result in an increased therapeutic window. On the other hand, if a combination would work exclusively synergistic in a specific tumor cell population (*e.g.*, *BRAF*-mutant cells) and not in other cells (*i.e.*, *BRAF*-wild type, or non-malignant cells), this would amplify the specific effect of the inhibitors. In parallel with the term 'targeted' for single agents, these combinations could be called 'targeted combinations.'

Here, we present novel drug combinations, specifically targeted at Wnt-pathway, *KRAS* or *MYC*-dependent cell growth. The finding is based on evaluating synthetic lethal effects after screening of a drug library in a panel of forty-four cancer cell lines. More than hundred new and established drugs, clinical and pre-clinical compounds have been characterized on this panel, which is being used to identify novel genomic drug sensitivity markers for anti-cancer drugs [31]. We have used this data set to select compounds that specifically inhibit cell lines carrying undruggable oncogenic drivers. A subsequent study of the synergistic interactions between these cellularly selective single agents, resulted in several novel drug combinations that target these 'difficult-to-drug' cancer genes. This demonstrates that it is possible to more optimally exploit the vulnerabilities of cancer cells by drug combinations, thereby increasing the efficacy of targeted therapy.

Materials and Methods

Compounds

All reference inhibitors and cytostatic agents were obtained from commercial vendors (overview in S1 Table). Dry powders of reference compounds were stored as indicated by the supplier. Before testing, compounds were dissolved in dimethyl sulfoxide (DMSO) and diluted in 20 mM sterile Hepes buffer pH 7.4, before addition to microtiter plates with cells [31].

Cell proliferation assays

All cell lines were purchased from the American Type Culture Collection (ATCC) (Manassas, VA, U.S.A.) (S2 Table) and cultured in the media as recommended by ATCC. All cells used were within nine passages of the original ATCC vial. Proliferation assays were carried out as described before [31] in 384-well plates with incubation with compound for 72 hours. Cell number was optimized for each cell line, to maximize assay window and to ascertain that growth was not limited by cell density. Compound effects were measured in a 9-point dilution series in duplicate. The final DMSO concentration during incubation was 0.4% (v/v) in all wells. As readout, we determined intracellular ATP content as an indirect measure of cell number, using ATPlite 1 Step solution (Perkin Elmer, Groningen, The Netherlands). The effect of the compounds on cell growth was calculated relative to control wells containing only 0.4% (v/v) DMSO. IC_{50}s were fitted by non-linear regression using XLfit5. Maximum and minimal signals were locked, where appropriate, to obtain the best fit as indicated by the *F*-test as implemented in XLfit5. If IC_{50}s did not fit within the tested concentration range, compounds were retested after further dilution.

Curve shift synergy experiments

For all synergy experiments, cell line proliferation assays were performed as outlined above. For curve shift analysis, compound stocks were diluted in DMSO on the day of the experiment, to concentrations of 3160 times their IC_{50}, as determined in the single agent experiments (S3 Table). Both solutions were subsequently mixed in three volume ratios (1:1, 1:4 and 4:1, unless

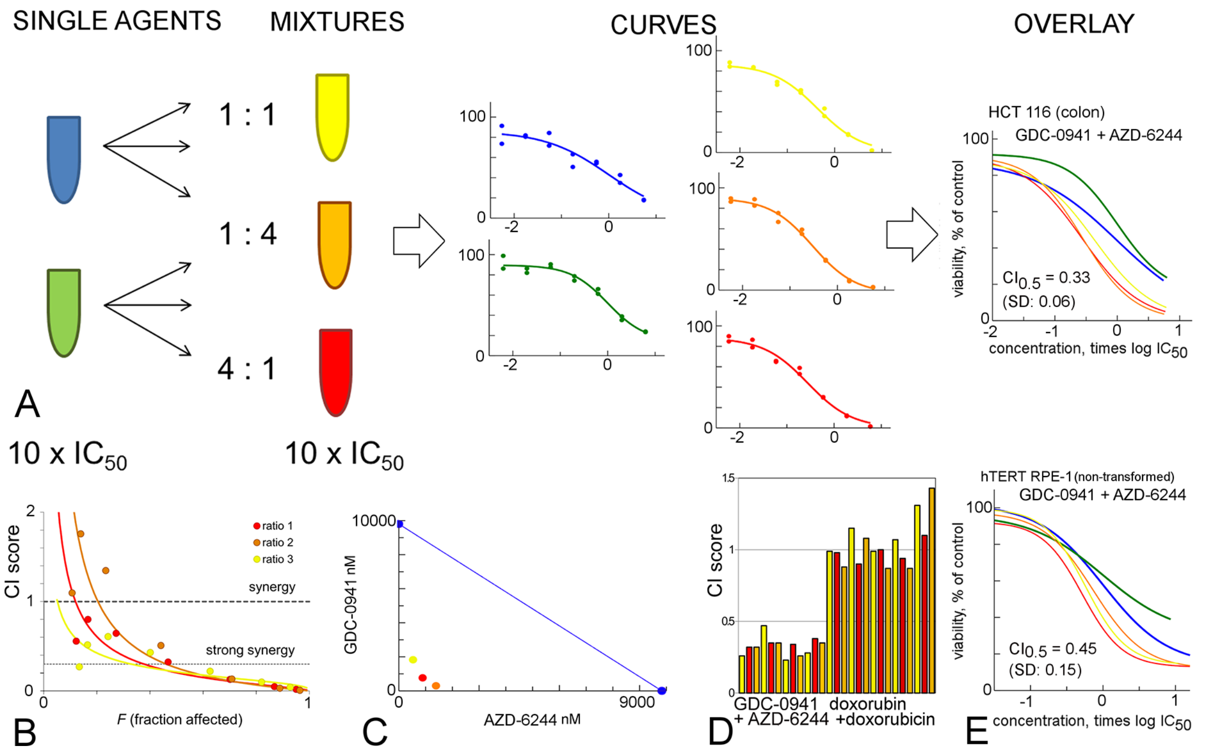

Fig 1. Setup of the curve shift synergy experiments. A: First, concentrated mixtures are made of the single agents. Subsequently, dose response curves are generated. For the overlay, all concentrations are scaled relative to the measured IC_{50}s of the single agents. The example shown is the effect of the PI3-kinase inhibitor GDC-0941 (green) and the MEK inhibitor AZD-6244 (blue) on proliferation of the HCT 116 cell line. Mixture ratios used were 1:1, red; 4:1, orange; 1:4, yellow. **B**: Calculation of the Combination Index (CI). The fraction affected *(F)* is equivalent to 1/100 of the %-effect. If CI < 1, compounds show synergy. The fitted CIs at *F* = 0.5 (50% effect), for all mixtures, are reported as $CI_{0.5}$. **C**: Calculation of the isobologram [7]. Single agent concentrations needed to achieve 75% effect in the cell proliferation assay are displayed in blue dots and connected by the blue line. The concentrations where the combination curves achieve 75% growth effect are displayed in red, yellow and orange, where the x and y coordinates are the respective component concentrations. If the combination points lie below the blue line, there is synergy. **D**: Reproducibility of $CI_{0.5}$ measurements in a positive control of AZD-6244 / GDC-0941 (light bars, average 0.33, SD: 0.06, n = 12) and a negative control of doxorubicin / doxorubicin (dark bars, average 1.04, SD: 0.16, n = 15). Both were combined in the HCT 116 cell proliferation assay. Every bar represents an individual mixture ratio (yellow, red, orange) that was tested in duplicate. **E:** Curve shift experiment of the AZD-6244 / GDC-0941 combination in the RPE-1 fibroblast cell line, which is immortalized using hTERT. Here, the combination also shows synergy.

doi:10.1371/journal.pone.0125021.g001

otherwise indicated, Fig 1) [8, 32]. Because of the IC_{50} matching, all stock solutions and mixtures will be equipotent when there is no synergy or antagonism between the two agents tested. The stocks of the mixtures and single agents were further diluted in DMSO to generate a 7-points dose-response series in duplicate. The final DMSO concentration during incubation was 0.4% (v/v) in all wells. Final assay concentrations range, for the single agents, between 10 and 0.01 times their IC_{50} (10 and 0.01 IC_{50} equivalents). For the single agent curves, IC_{50}s were fitted on the %-effect data by non-linear regression using XLfit5. To correct for inter-assay variation, the IC_{50}s of the single agents in the synergy experiment were used to calculate the total mixture potencies. If, for instance, in the final synergy experiment a single agent potency was 1.2 times higher than expected, all x-axis values along the dilution curve were divided by 1.2 (Fig 1A). This ensures proper overlay of all curves in the experiment.

From the same data, Combination Indices (CI) were determined according to the method of Chou and Talalay [8]. Briefly, from the fitted curve parameters, the concentrations of components A and B that induce 50% cell viability were determined, both for the single agent and mixtures experiments. Then, $CI_{0.5,\ mix1}$ = [compound A]$_{mix1}$ / [compound A]$_{single}$ + [compound B]$_{mix1}$ / [compound B]$_{single}$. CI values were calculated in Microsoft Excel, using the

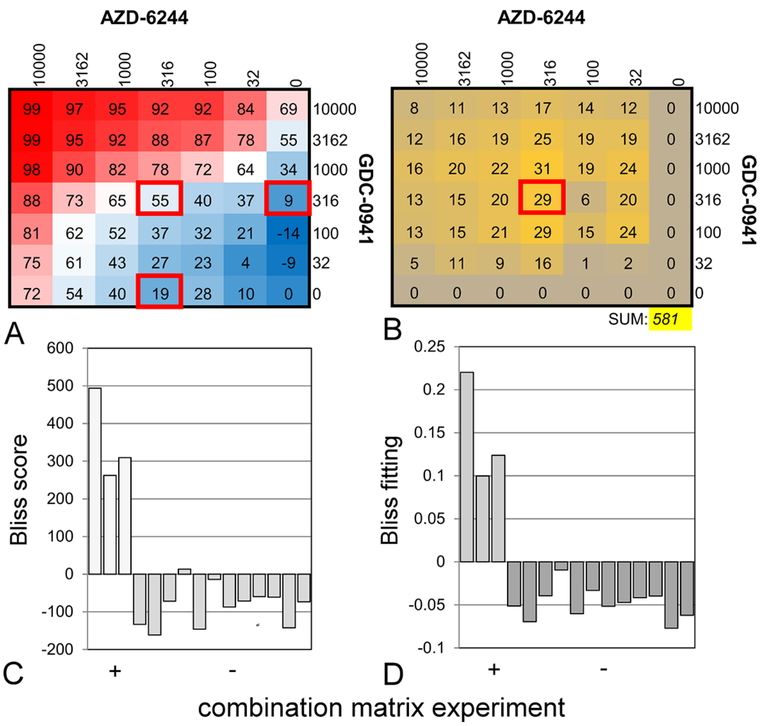

Fig 2. Setup of a combination matrix experiment, which is an alternative to a curve shift experiment. Dilution series of the MEK inhibitor AZD-6244 (rows, in nM) and the PI3K inhibitor GDC-0941 (columns, in nM) were mixed and their effect was measured in the HCT 116 cell line proliferation assay. **A**: Relative inhibitory effects (%) in the assay. **B**: Bliss scores calculated by relating the mixture effects to the single agent effects, using the following procedure, illustrated by the data in red squares: 1. the %-inhibition effects are converted to fractional growth effects by 1 - (%-inhibition / 100). For the indicated squares, the values become 0.45, 0.91 and 0.81, respectively; 2. The Bliss score is then equal to the observed effect for the mixture, with the additive effects of the single agents subtracted. This amounts to 0.45 - (0.91 · 0.81) = 0.29; 3. This leads to the value of 29% indicated in the right panel. The total Bliss score in yellow shading is the summation of all wells [4, 33]. **C**: Reproducibility of the combination matrix screening. The experiment was carried out using the same reagents as in Fig 1D. Positive controls are in white (average 355, SD: 122, n = 3), negatives in grey (average -84, SD: 54, n = 12). Bliss scoring was performed as outlined under panel B. **D**: as panel C, where the outcome is evaluated with a Bliss parameter fitting protocol to smoothen assay noise [5] (S1 Equation, positive controls in light grey (average 0.14, SD: 0.06, n = 3), negatives in dark grey (average -0.05, SD: 0.02, n = 12).

doi:10.1371/journal.pone.0125021.g002

same curve parameters that were used in evaluation of the curve shift. In cases with low efficacy curves, the program Calcusyn was used [8]. Because this software refits curves, it was not our method of preference. Each experiment contained three mixture ratios (1:1, 4:1, 1:4) in duplicate. Synergy experiments were repeated at least three times, on separate occasions, unless otherwise indicated (S4 Table). The three mixtures and three replicates (or more) resulted in at least nine independent $CI_{0.5}$ values, from which averages and standard deviations were calculated. As further graphical check, isobolograms were generated in Microsoft Excel (*i.e.* Figs 1–11) [7].

Combination matrix synergy experiments

To determine synergy with the combination matrix method [4–6], cell proliferation assays were performed as described above. At the day of the experiment, two single agents stocks were serially diluted in a 6-point series in either a row or a column (Fig 2A). A 7th point was filled with 100% DMSO without compound. Subsequently, the complete 7 x 7 combination matrix

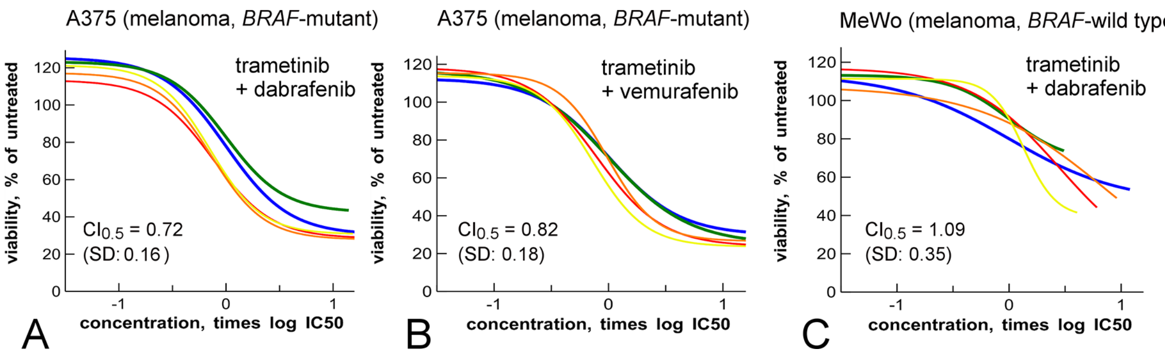

Fig 3. Targeted synergy between MEK- and BRAF-inhibitors. A: Curve shift experiment of trametinib (blue) and dabrafenib (green) in a proliferation assay of the *BRAF*-mutant A375 cell line. Yellow, red, orange are mixture curves as described in Fig 1. **B**: as panel A but with vemurafenib (green) as BRAF inhibitor. **C**: As panel A, but using proliferation assay of the *BRAF*-wild type melanoma MeWo cell line. Note that in panels A and B, there is synergy, although more in A than in B. In panel C, there is no synergy, as judged by CI and efficacy. The high variation for the CI in panel C is caused by the partial efficacy of all curves.

doi:10.1371/journal.pone.0125021.g003

was generated by 1:1 mixing of all single agent concentrations. Final tested compound concentrations range between 3 nM—1000 nM. The final DMSO concentration during incubation was 0.4% in all wells. The %-effect data from the proliferation assays were used to calculate an excess over Bliss additivity score, which was subsequently summed over the total combination matrix to generate a total Bliss score [4, 33] (Fig 2B). Alternatively, a Bliss model of compound independence was minimized on the %-effect data by adjusting a parameter β that is 1.0 for total additivity, and higher in case of synergy (S1 Equation) [5].

Analysis of cell panel response data

The IC_{50} from the cell proliferation assays were used to calculate a selectivity parameter for each compound, the selectivity entropy (s_{sel}) [34]. The s_{sel} was used to rank compounds for their cellular selectivity (Fig 5).

Anova analysis was used to determine whether there is a statistical correlation between drug sensitivity and the genotype of the cancer cell lines used in the proliferation assays. Cancer gene mutation status of the cell lines was downloaded from the Cancer Cell Line initiative (CCL, S2 Table) [9]. IC_{50} data were correlated to cell line genotype by type II Anova in the

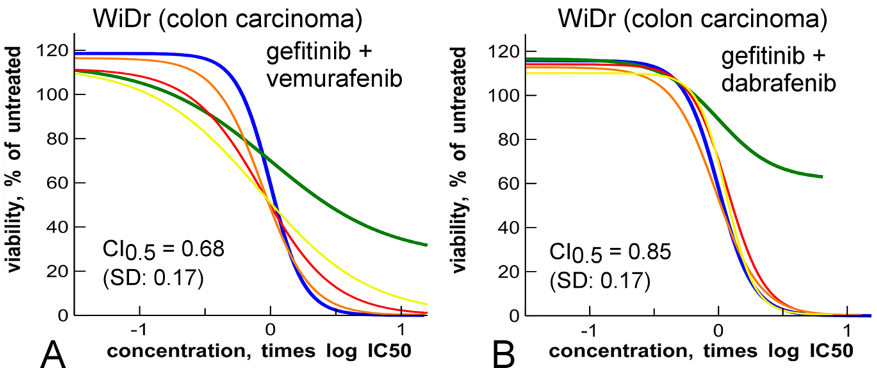

Fig 4. Synergy between EGFR and BRAF inhibitors in a colon carcinoma line. A: Curve shift experiment of the EGFR inhibitor gefitinib (blue) and the BRAF inhibitor dabrafenib (green) in a proliferation assay of the *BRAF*-mutant WiDr colon carcinoma cell line. Yellow, red, orange are mixture curves as described in Fig 1. **B**: as panel A but with vemurafenib (green) as BRAF inhibitor. In panel A, there is clear synergy between the two drugs, in panel B, there is less synergy.

doi:10.1371/journal.pone.0125021.g004

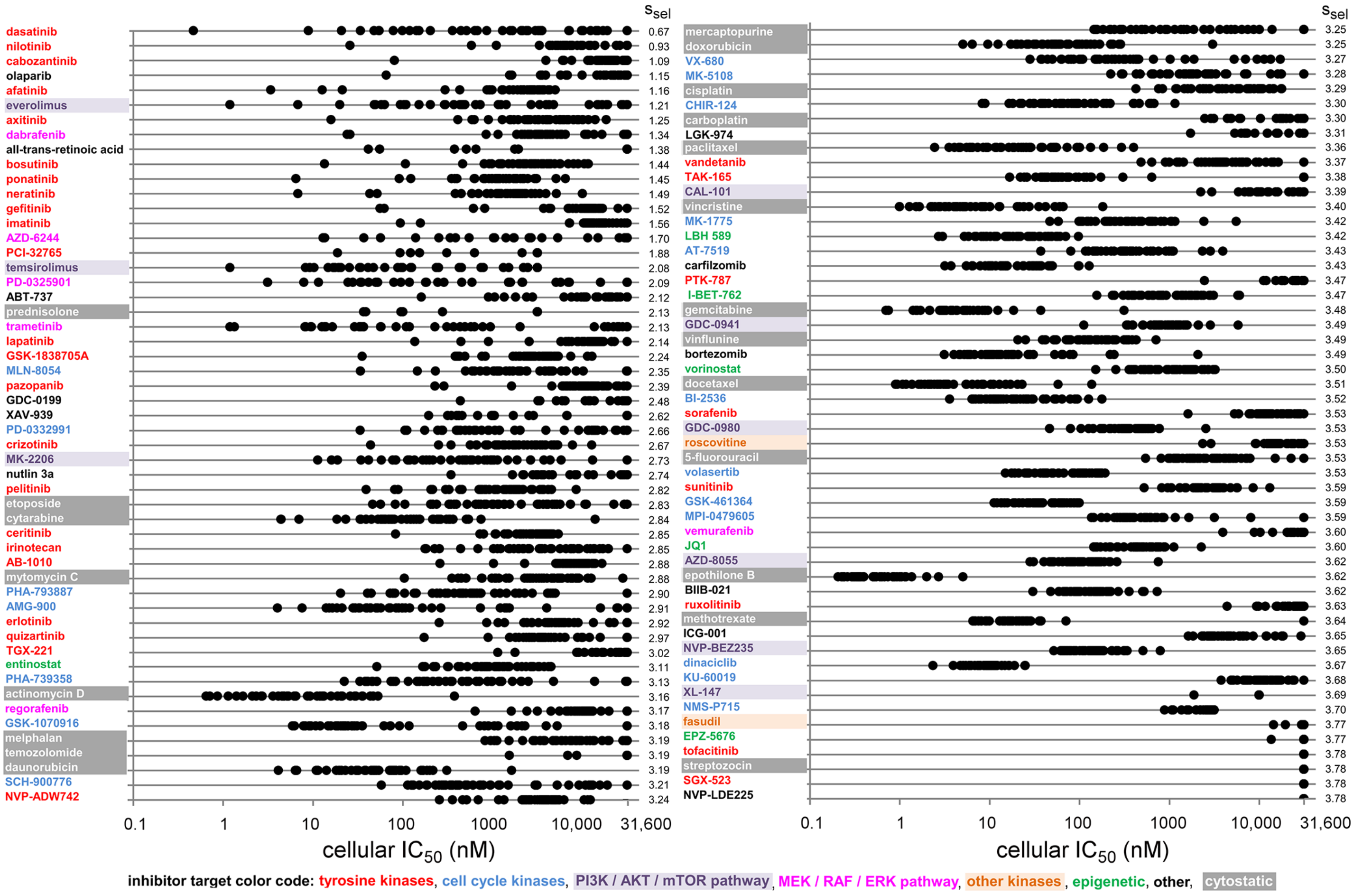

Fig 5. Distribution of IC_{50}s after profiling of a drug library on a 44-cell line panel. Compound effects were measured in dose-response in parallel on forty-four different cell line proliferation assays. IC_{50}s were fitted. Every black dot represents the IC_{50} of a separate compound and cell line combination. Each line contains forty-four IC_{50}s. Compounds are ordered according to their cellular selectivity, as indicated by an entropy value (s_{sel}) that is based on a Boltzmann-weighted average of IC_{50} values [34]. At the top-left are the most selective compounds in the cell proliferation assays, *i.e.*, the compounds that gave the most differentiated effects in the proliferation assays across the 44-cell line panel. The least selective drugs, which gave the least differentiated effects across cell lines, are indicated bottom-right. Names of the inhibitors are colored according to their biological mechanism.

doi:10.1371/journal.pone.0125021.g005

program R [35], Significance was evaluated after multiple testing correction of the p-values, and the results were depicted in volcano plots, according to a method outlined in earlier work [31] (Fig 6).

To evaluate if an inhibitor is significantly more potent in cell lines with particular genetic changes, a one-sided t-test was carried out in Microsoft Excel (Fig 7). This tested if IC_{50}s in the group of oncogene-carrying cell lines were significantly lower than those in wild type cell lines (for cell line genotypes, see S2 Table, for individual IC_{50}s, see S3 Table).

Compound selection for combination studies

Based on the cell line response data, compounds were selected for combination testing. The underlying hypothesis was that compounds that work in similar oncogenic backgrounds, have a higher chance of forming synergistic pairs, providing these compounds have different biological mechanisms. High-ranking (in Fig 7) inhibitors of proliferative pathways such as RAS, EGFR or ERBB2 were first selected: trametinib for *CTNNB1*, TAK-165 for *KRAS*, neratinib for

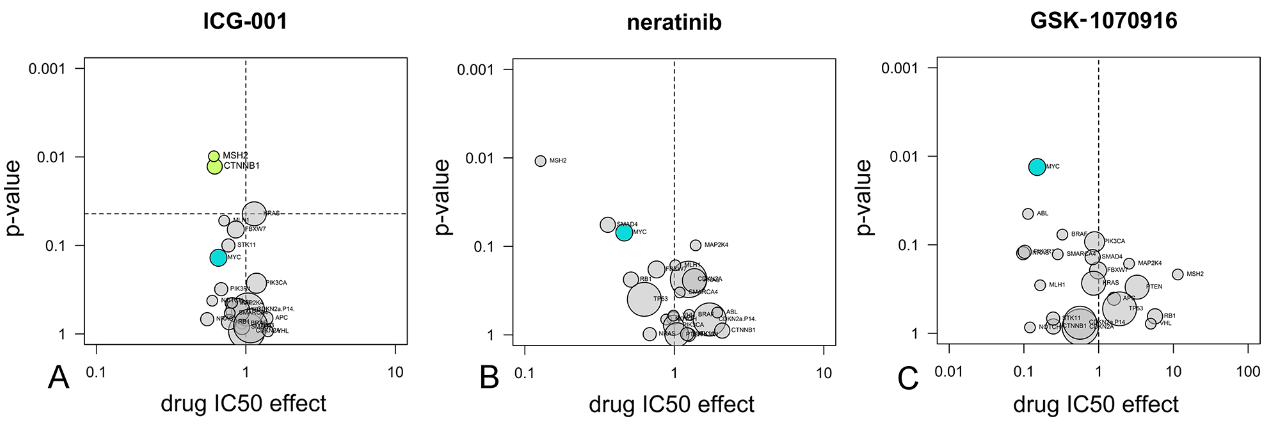

Fig 6. Anova analysis to reveal drug sensitivity markers. The sensitivity of drugs for twenty-three common cancer gene mutations was analysed statistically through Anova, based on the IC_{50} data on forty-four cell lines, and depicted in volcano plots. On the x-axis: IC_{50} shift between wild type and mutant cell lines. On the y-axis: p-value for significance in the Anova variance test. Significance was corrected for multiple-testing and all associations above the threshold (dotted line) were colored green [31]. In panels B and C no associations are above the multiple testing threshold and therefore no dotted line is shown. Areas of circles are proportional to the number of cell lines carrying mutations. **A**: The Wnt-pathway inhibitor ICG-001 [39] is relatively potent in cell lines containing *CTNNB1* or *MSH2* mutations. **B**: the EGFR inhibitor neratinib (HKI-272) [61] is relatively potent in cell lines containing *MSH1*, *SMAD4* mutations, or *MYC* amplifications (blue). **C**: the Aurora inhibitor GSK-1070916 [62] is relatively potent in cell lines containing *MYC* amplifications (blue) or *ABL* translocations.

doi:10.1371/journal.pone.0125021.g006

MYC. These were combined with high ranking inhibitors from other classes, *i.e.* cell cycle inhibitors or taxanes. Preference was given to partner compounds that showed significant association in Anova ($p < 0.05$) or that showed significance in the t-test ($p < 0.05$) combined with

	CTNNB1			*KRAS*			*MYC*	
compound	***log ΔIC50***	***p-value***	***compound***	***log ΔIC50***	***p-value***	***compound***	***log ΔIC50***	***p-value***
trametinib	-1.57	0.005 [1,2]	TAK-165	-0.82	0.001 [1]	GSK-1070916	-0.82	0.0001 [1,2]
PD-0325901	-1.56	0.014	PD-0325901	-0.62	0.082	paclitaxel	-0.45	0.003 [1,2]
AZD-6244	-1.04	0.073	trametinib	-0.61	0.114 [1]	GSK-1838705A	-0.39	0.167
AMG900	-1.03	0.005	AZD-6244	-0.58	0.062	AZD-6244	-0.38	0.303
TAK165	-0.81	0.425	NMS-P715	-0.55	0.089 [2]	BI-2536	-0.38	0.025 [1,2]
MLN-8054	-0.77	0.078	AMG-900	-0.40	0.104	MLN-8054	-0.34	0.005
PD-0332991	-0.76	0.014	cytarabine	-0.39	0.041	neratinib	-0.33	0.270 [1]
GSK-1070916	-0.61	0.003	MLN-8054	-0.37	0.009 [1]	docetaxel	-0.33	0.023 [1,2]
nutlin 3a	-0.46	0.002	MPI-0479605	-0.28	0.001	AMG-900	-0.31	0.301
GSK-1838705A	-0.45	0.272	melphalan	-0.26	0.073	PCI-32765	-0.31	0.284
MK-2206	-0.44	0.188	nutlin3a	-0.21	0.100	XAV-939	-0.30	0.209
XAV-939	-0.41	0.339	etoposide	-0.20	0.206	melphalan	-0.29	0.013
MK-5108	-0.37	0.111	entinostat	-0.19	0.126	GSK-461364	-0.29	0.020
cytarabine	-0.31	0.084	crizotinib	-0.18	0.075	PD-0332991	-0.28	0.439
AT-7519	-0.30	0.003	MK-5108	-0.17	0.135	vorinostat	-0.24	0.011
vincristine	-0.22	0.102	GSK-1838705A	-0.14	0.205	irinotecan	-0.23	0.083
ICG-001	-0.21	0.003 [1,2]	BIIB-021	-0.13	0.063	afatinib	-0.23	0.326
doxorubicin	-0.20	0.011	sorafenib	-0.12	0.073	cytarabine	-0.23	0.174
irinotecan	-0.20	0.258	methotrexate	-0.12	0.171	methotrexate	-0.23	0.051
docetaxel	-0.19	0.023	MK-2206	-0.11	0.403	lapatinib	-0.23	0.304
daunorubicin	-0.19	0.102	nilotinib	-0.09	0.247	pazopanib	-0.18	0.164

Fig 7. Compounds that most specifically inhibit *CTNNB1*- or *KRAS*-mutant, or *MYC*-amplified cell lines. Compound names were colored according to inhibitor class, analogous to Fig 5. **Δ log IC_{50}** indicates the ^{10}log of the ratio between average IC_{50}s in the wild type and mutant (amplified) cell lines. A horizontal line is drawn at -0.3, indicating a $10^{-0.3} = 0.501$ shift in IC_{50}, making the compound $^{1}/_{0.501} \approx 2$ times more potent on mutant (amplified) cell lines. The forty-four cell line panel comprised four *CTNNB1*-mutants, ten *KRAS*-mutant and five *MYC*-amplified cell lines from a variety of tissue origins (see S2 Table). The **p-value** is calculated from a t-test, to validate if IC_{50}s in mutant (amplified) cell lines are significantly lower than in wild type cell lines. [1]compound selected for follow-up in synergy studies. [2] significant association in the drug sensitivity Anova analysis that tested 23 genes simultaneously.

doi:10.1371/journal.pone.0125021.g007

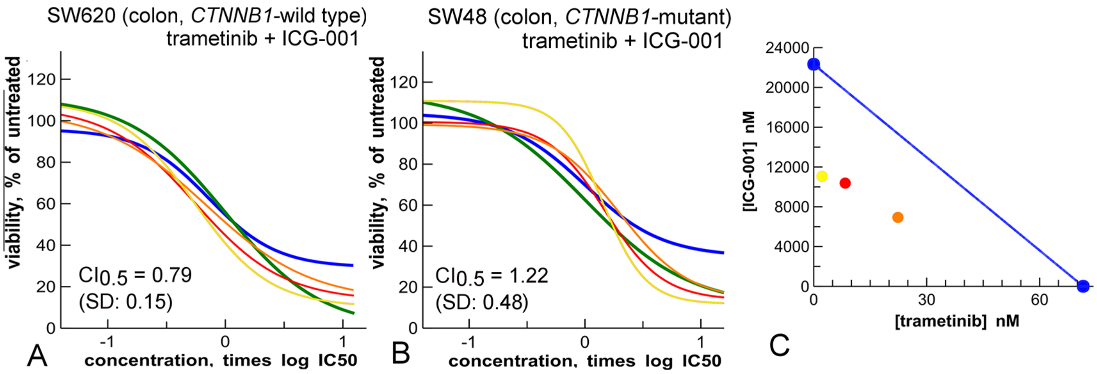

Fig 8. Synergy of the MEK inhibitor trametinib (blue) and the Wnt-pathway inhibitor ICG-001 (green) in colon cancer cell lines. A: Synergy in *CTNNB1*-wild type cell line SW620. This cell line is nevertheless a Wnt-pathway-mutant as it is mutated in *APC*. In addition, SW620 is *TP53*-mutant. **B:** Absence of synergy in the *CTNNB1*-mutant cell line SW48. This cell line is *TP53*-wild type**. C:** isobologram of results in panel A, at the 75% effect cut-off level. Yellow, red, orange are mixture curves as described in Fig 1.

doi:10.1371/journal.pone.0125021.g008

reasonable IC_{50} differences ($logIC_{50} < -0.3$, a factor 2). The selected compounds are shown in Table 1; individual IC_{50}s in S3 Table. For *MYC*, the tyrosine kinase inhibitor neratinib was preferred over GSK-1838705A because neratinib is cellularly more active and selective. For *KRAS*, trametinib was chosen as additional partner to TAK-165, although $p > 0.05$, because it is known that MEK inhibitors are active in KRAS-mutant cell lines [36].

Results

Curve shift is the most robust setup to observe synergy

Much research has gone into defining the best way of determining synergy between two compounds in an *in vitro* assay. From this, two basic methods have evolved, the curve shift analysis (Fig 1) [8,32] and the combination matrix experiment with Bliss-scoring (Fig 2) [4–6]. We first performed robustness experiments to determine the optimal experimental setup.

For curve shift analysis, equipotent stock solutions of two drugs are mixed in various ratios and dose-response curves are determined. In case of synergy, the mixture curves are shifted leftward (Fig 1A). In case of antagonism, the mixture curves are shifted rightward. From the same experiment we calculated the Combination Index (CI) to quantify synergy (Fig 1B) [8],

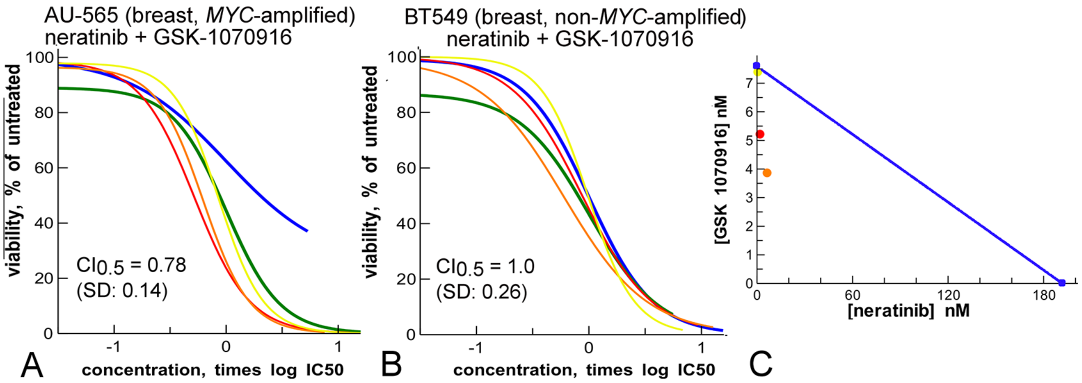

Fig 9. Synergy of neratinib (blue) and GSK-1070916 (green) depends on *MYC*-amplification. A: amplified cell line**. B:** non-amplified cell line**. C:** isobologram of results in panel A, at the 75% effect cut-off level. Yellow, red, orange are mixture curves as described in Fig 1. For panel B, 100% effect levels were set to the effect at the highest tested concentration.

doi:10.1371/journal.pone.0125021.g009

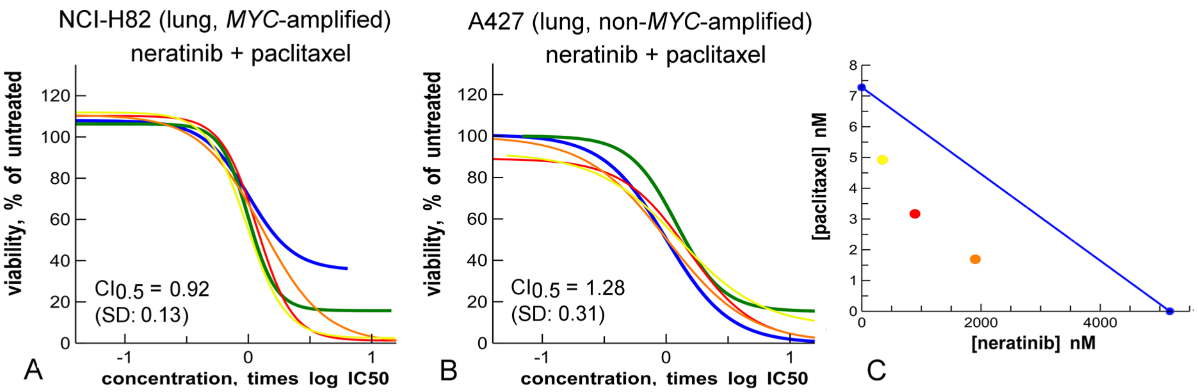

Fig 10. Synergy of neratinib (blue) and paclitaxel (green) in cells containing a *MYC* amplification. A: *MYC*-amplified. **B:** non-*MYC*-amplified. **C:** isobologram of results in panel A, at the 75% effect cut-off level. Yellow, red, orange are mixture curves as described in Fig 1.

doi:10.1371/journal.pone.0125021.g010

and visualized synergy in an isobologram (Fig 1C). The curve shift method can be used to determine which concentration is necessary to achieve a predetermined effect, *e.g.*, 50% of maximum effect. This is known as the Loewe concept of synergy [37].

We validated the curve shift method by testing the combination of the PI3K inhibitor GDC-0941 and the MEK inhibitor AZD-6244 in colon carcinoma HCT 116 cells (Fig 1), a synergistic combination previously described by Haagensen *et al.* [38] following a biological discovery by Liu *et al.* [27]. The $CI_{0.5}$ value of GDC-0941/AZD-6244 is 0.33, indicating strong synergy. In addition, the curve overlays show that at the highest concentrations, the combination also has increased efficacy compared to the single agents (Fig 1A).

To examine robustness, we repeatedly tested the GDC-0941/AZD-6244 combination, and the neutral combination of doxorubicin with doxorubicin (Fig 1D). The CI values of GDC-0941/AZD-6244 (average: 0.33, standard deviation (SD): 0.06, n = 12, Fig 1D) are well reproducible across experiments and across mixtures. The neutral combination has a CI of about 1, as theoretically expected (average: 1.04, SD: 0.16, n = 15, Fig 1D). This shows that the curve shift method can reliably detect synergy.

Next we evaluated the combination matrix method. Here, dilution series of two compounds are mixed along the rows and columns of a matrix (Fig 2A). The signal in each well is scored using the Bliss additivity criterion (Fig 2B) [4, 33]. Because changes in the relative (%) effect at predetermined compound concentrations are calculated, the Bliss approach is fundamentally

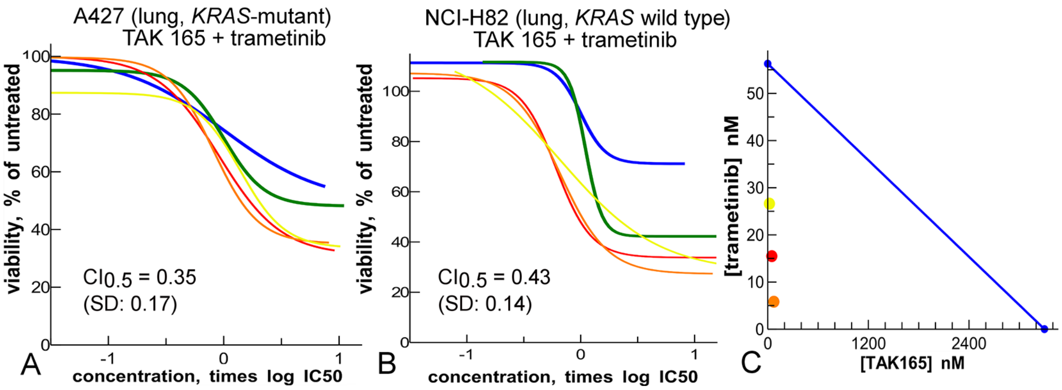

Fig 11. Synergy of the EGFR/ERBB2 inhibitor TAK-165 (blue) and the MEK inhibitor trametinib (green) is independent of *KRAS*-status. A: *KRAS*-mutant. **B**: *KRAS*-wild-type. **C:** isobologram of results in panel A, at the 50% effect cut-off level. Yellow, red, orange are mixture curves as in Fig 1.

doi:10.1371/journal.pone.0125021.g011

Table 1. Compounds selected for synergy studies.

genetic driver	compounds in combination set	biochemical target	IC_{50} shift	p-value[1]	p-value[2]
BRAF	Dabrafenib	BRAF kinase	284	1E-29	7E-6
**mutation**	Trametinib	MEK kinase	35	2E-8	0.2
	vemurafenib	BRAF kinase	3.0	0.2	4E-4
CTNNB1	Trametinib	MEK kinase	37.0	0.005	0.02
**mutation**	ICG-001	Wnt-pathway	1.6	0.003	0.01
MYC	neratinib	EGFR kinase	2.2	0.3	0.07
**amplification**	GSK-1070916	Aurora B/C kinase	6.6	0.0001	0.01
	BI-2536	PLK1 kinase	2.4	0.03	6E-3
	paclitaxel	spindle poison	2.8	0.003	0.008
	docetaxel	spindle poison	2.1	0.02	0.01
KRAS	TAK-165	ERBB2 kinase	6.6	0.001	0.3
**mutation**	trametinib	MEK kinase	4.1	0.1	0.9
	MLN-8054	Aurora A/B kinase	2.3	0.009	0.3

[1]Significance as determined by a one-gene t-test.

[2]Significance as determined by the 23-gene Anova analysis, before multiple testing correction. A p-value < 0.05 is considered statistically significant.

doi:10.1371/journal.pone.0125021.t001

different from the Loewe approach [37]. Also with the combination-matrix method, the combination of GDC-0941 and AZD-6244 showed synergy (Fig 2C). However, the variation between different experiments was significantly higher than in experiments where the curve shift method was used (average: 355, SD: 122, n = 3, Fig 2C). Because this variation could in part be due to absence of data fitting, we implemented an algorithm in which a synergy parameter β is fitted to the formula for Bliss additivity (see Methods and Fig 2D) [5]. This did not show better reproducibility (average: 0.14, SD: 0.06, n = 3, compare Figs 1D and 2D). Interestingly, the Bliss methods had the tendency to assign antagonism to the control of doxorubicin with itself.

Based on the data, we evaluated signal to noise ratios (average/SD) for the three synergy metrics. The GDC-0941/AZD-6244 combination experiment has a ratio of 5.5, 2.9 and 2.3 for curve shift, Bliss scoring and Bliss fitting methods, respectively. For the doxorubicin with doxorubicin control, these ratios are 6.5, 1.5 and 2.5, respectively. Both sets of values indicate that the curve shift has the best signal to noise ratio.

Synergy between GDC-0941 and AZD-6244 in non-malignant cells

The PI3K-inhibitor GDC-0941 and the MEK inhibitor AZD-6244 are both targeted compounds in development for oncology, and show potent synergy (Fig 1A) However, since PI3K and MEK play a central role in regulating cell growth, these compounds also inhibit normal proliferating cells and thus exhibit significant toxicities [39]. If the synergy between the two compounds would be limited to malignant cell lines, this would provide new opportunities for their application as cancer therapy. For this reason we tested for synergy between GDC-0941 and AZD-6244 in two models of normal proliferating cells: human telomerase (hTERT)-immortalized cells derived from human retinal epithelia (hTERT RPE-1) and human foreskin fibroblasts (BJ-5ta) that also have been immortalized with hTERT. GDC-0941 inhibits the proliferation of these cell lines with IC_{50} of 760 nM and 1.1 μM, respectively. AZD-6244 inhibits their proliferation with IC_{50} of 91 and 94 nM. In the hTERT RPE-1 cell line, the combination works synergistically ($CI_{0.5}$ = 0.45, SD: 0.15, n = 15, Fig 1E). Synergy was also found in the BJ-5ta cell line ($CI_{0.5}$ = 0.43, SD: 0.09, n = 16, S1 Fig). This demonstrates that the synergy between GDC-0941 and AZD-6244 is not specific to cancer cells.

Curve shift shows that trametinib and dabrafenib are a targeted drug combination

Next, we used the curve shift method to study the combination of the MEK inhibitor trametinib and the BRAF inhibitor dabrafenib, which has shown synergistic activity in *BRAF*-mutant melanoma cell lines [28], and in melanoma patients [40]. As single agents, dabrafenib and trametinib are potent inhibitors of the proliferation of the *BRAF*-mutant A375 melanoma cell line, with IC_{50} of 26 nM and 19 nM, respectively [31]. Curve shift assays confirmed synergy of the two compounds (Fig 3A). We also examined the combination of trametinib with vemurafenib, another BRAF inhibitor (Fig 3B). As single agent vemurafenib has an IC_{50} of 4 μM in the A375 cell line assay. Although the curve shift indicates that the combination of trametinib and vemurafenib is synergistic, the CI was higher (*i.e.*, $CI_{0.5}$ = 0.82, SD: 0.18, n = 15) than for the combination of trametinib and dabrafenib ($CI_{0.5}$ = 0.72, SD: 0.16, n = 15, Fig 3).

Because dabrafenib and trametinib can also inhibit the growth of non-*BRAF*-transformed cells [31], we investigated if their synergy also occurs in the *BRAF*-wild type MeWo melanoma cell line. Both dabrafenib and trametinib inhibit the proliferation of this cell line with IC_{50}s of 987 nM and 46 nM, respectively (Fig 3C). However, both compounds show only partial efficacy (Fig 3C), which is in line with the absence of an oncogenic *BRAF*-mutation in this cell line [9]. When mixed, the combination does not show synergy in the form of a curve shift or increased efficacy ($CI_{0.5}$ = 1.09, SD: 0.35, n = 9, Fig 3C). In the non-transformed hTERT RPE-1 and BJ-5ta cell lines, the combination is even antagonistic (S2 Fig). From this, we conclude that the combination of dabrafenib and trametinib is specifically targeted to oncogenic BRAF-signaling.

Curve shift analysis of EGFR and BRAF inhibition in a *BRAF*-mutant colon carcinoma line

Recently, it was suggested that the growth of *BRAF*-mutant colon carcinoma cell lines can be inhibited by combining a BRAF inhibitor with an EGFR inhibitor, which both were suggested to be inactive as single agents [29]. As this seemed an interesting example of a synergistic interaction that only takes place in a particular cancer cell type (*i.e. BRAF*-mutant, EGFR overexpressing colon cancer cell lines), we performed curve shift assays with the EGFR inhibitor gefitinib and the BRAF inhibitors vemurafenib and dabrafenib, in proliferation assays with the *BRAF*-mutant colon carcinoma WiDr cell line (Fig 4). Although we can confirm the poor single agent activities of gefitinib (IC_{50} = 8 μM), vemurafenib as well as dabrafenib show a clear dose-response curve in the WiDr cell proliferation assay, with IC_{50}s of 900 nM and 76 nM, respectively (Fig 4). At the highest concentrations, the BRAF inhibitors have only partial efficacy as single agents, whereas in the combinations, when mixed with a relatively small amount of EGFR inhibitor (such as in the dose ratio of 4:1), full efficacy is obtained (Fig 4). Although the mixture curves are not shifted leftward, the CI values indicate synergy, particulary for gefitinib and vemurafenib (Fig 4).

Cell panel profiling of a drug library

Having demonstrated that our method can identify targeted drug combinations, we proceeded with identifying novel combinations targeted at difficult-to-drug cancer genes. We used a proprietary data set, consisting of the activities of more than hundred small molecule compounds in proliferation assays of forty-four human cancer cell lines, to identify compounds targeting major cancer drivers. The compounds include twenty-eight marketed cytotoxic drugs, all

FDA-approved small molecule kinase inhibitors, and many experimental targeted agents, such as modulators of epigenetic targets and experimental kinase inhibitors (Fig 5, S1 Table).

The IC_{50} values of the compounds across cell lines are summarized in Fig 5, where compounds are ranked according to the selective response they display in the cell panel, which is quantified using selectivity entropy (s_{sel}). The s_{sel} is a single value summary of the selectivity of each inhibitor, and is high if all cellular IC_{50}s are equal (aselective), and low if only one cell line is inhibited potently (selective response) [34]. As expected, the most differentiated profiles, with low entropy values, are obtained from targeted agents, in particular several marketed kinase inhibitors (Fig 5). The ABL inhibitors dasatinib (s_{sel}, 0.67) and nilotinib (s_{sel}, 0.93), and the growth factor kinase inhibitor cabozantinib (s_{sel}, 1.09) are the most selective from all compounds tested (Fig 5). The least differentiated responses, with high entropy values, are from classic cytotoxic agents, such as epothilone B (s_{sel}, 3.62), and inhibitors of general cellular processes, such as cell cycle inhibitors and inhibitors of the PI3K pathway (Fig 5). Also remarkable is that a number of cytotoxic agents, such as the DNA synthesis inhibitor cytarabine (s_{sel}, 2.84). and the topoisomerase II inhibitor etoposide (s_{sel}, 2.83), are comparable to certain targeted inhibitors in terms of cellular selectivity (*cf*. the irreversible EGFR inhibitor pelitinib, s_{sel}, 2.82, or the c-Kit inhibitor AB1010 (masitinib), s_{sel}, 2.88). Inhibitors of the upcoming target class of epigenetic modulators, such as the bromodomain inhibitors JQ1(s_{sel}, 3.60) and I-BET-762 (s_{sel}, 3.47), and the histone deacetylase (HDAC) inhibitors entinostat (s_{sel}, 3.11) and vorinostat ($s_{sel,}$ 3.50), show only moderate cellular selectivity.

Next, we combined the cellular IC_{50} data with genomic information of the cell line panel to search for drug sensitivity markers. We previously validated this approach with a number of clinically applied kinase inhibitor drugs [31]. Fig 6 demonstrates compounds that specifically inhibit cell proliferation driven by difficult-to-drug cancer genes. For example, ICG-001 was identified by high-throughput screening of a cell line expressing a β-catenin- (*CTNNB1)* dependent reporter gene [39]. Anova analysis of the profiling data from our cell line panel confirms that ICG-001 is more active in inhibiting the proliferation of cell lines that are mutated in *CTNNB1* compared to cell lines that carry the wild-type gene variant (Fig 6A). Another example is the EGFR/ERBB2 inhibitor neratinib, which, like other EGFR inhibitors [31], preferably inhibits the proliferation of cell lines with a mutation in *SMAD4* (Fig 6B). In addition, neratinib preferably inhibits cell lines with amplified *MYC* (Fig 6B). Also GSK-1070916, a potent inhibitor of Aurora B and C kinases, preferably inhibits the proliferation of cell lines with amplified *MYC* (Fig 6C).

Compounds targeting the *CTNNB1*, *KRAS* or *MYC* oncogenes

Given the high need to treat cancers driven by *CTNNB1*, *KRAS* and *MYC* oncogenes, we extended the Anova analyses of Fig 6 to all compounds tested, and ranked them on the basis of their relative sensitivities for cell lines expressing these cancer genes (Fig 7). We identified eight, five and one compounds with at least a 3.2-fold more potent IC_{50} for *CTNNB1*-mutant, *KRAS*- mutant or *MYC*-amplified cell lines, respectively (Fig 7). These shifts compare favorably with the 3-fold IC_{50} shift observed for the FDA-approved BRAF inhibitor vemurafenib in targeting *BRAF*-mutant cells (Table 1). A t-test was performed to assess if the IC_{50} shifts were significant ($p < 0.05$, Fig 7). Based on these results, compounds were chosen for combination studies (Table 1). Growth pathway inhibitors (such as MEK or tyrosine kinase inhibitors) were combined with compounds acting through other mechanisms, such as ICG-001, cell cycle inhibitors and taxanes (Fig 7). Preference was given to compounds that showed significant association in the Anova analysis ($p < 0.05$) or that showed significance in the t-test ($p < 0.05$) combined with at least 2-fold IC_{50} potentiation (Table 1).

Targeted combinations for *CTNNB1*-mutant cancers

As compounds that preferentially inhibit *CTNNB1*-mutant cell lines, our profiling identified the MEK inhibitor trametinib and the Wnt-pathway inhibitor ICG-001, both with high significance in the Anova test (Figs 6 and 7, Table 1). Particularly, greater than 10-fold IC_{50} shifts were seen for a series of MEK inhibitors (trametinib, PD-0325901 and AZD-6244) in *CTNNB1*-mutant lines (Fig 7).

Synergistic interactions between trametinib and ICG-001 were investigated in a panel of cell lines, including three *CTNNB1*-wild type colon cancer cell lines, three *CTNNB1*-mutant colon cancer cell lines, and a pair of *CTNNB1*-mutant and *CTNNB1*-wild type lung cancer cell lines (Fig 8 and S3 Fig). Interestingly, and in contrast to our expectations, trametinib and ICG-001 appear to work synergistically in the *CTNNB1*-wild type colon cancer cell line SW620 (Fig 8A), and not in the *CTNNB1*-mutant cell line (Fig 8B). This is confirmed by isobologram analysis (Fig 8C). To generate more reliable CI averages, the experiments were repeated seven times, on several occasions by different scientists (Fig 8, S4 Table). the result is supported by experiments in other cell lines. In the *CTNNB1*-wild type colon cancer cell line HCT 15 synergy is observed, and not in other lines such as HCT 116 (mutant), LoVo (wild type) and LS174T (mutant, S3 Fig). In lung cancer cell lines, the result is more straightforward. Here the combination of ICG-001 and trametinib shows synergy in the *CTNNB1*-mutant cell line (A427, $CI_{0.5}$ = 0.76, SD: 0.11) and not in the wild type cell line (A549, $CI_{0.5}$ = 1.03, SD: 0.06, S3 Fig).

Targeted combinations for *MYC*-amplified cancer

In the cell line panel, the EGFR/ERBB2 inhibitor neratinib, the Aurora inhibitor GSK-1070916, the PLK1 inhibitor BI-2536 and the taxane paclitaxel preferentially inhibit *MYC*-amplified cell lines (Fig 7, Table 1). The p-values from the t-test and the presence of inhibitors with similar mechanisms in the list of *MYC*-targeted compounds indicate that the effects are real (Fig 7). For instance, other Aurora inhibitors (MLN-8054 and AMG-900), another taxane (docetaxel), another PLK1 inhibitor (GSK-461364) and other EGFR/ERBB2 inhibitors (afatinib, lapatinib) are also among the *MYC*-targeted compounds (Fig 7).

We tested combinations of the EGFR/ERBB2 inhibitor neratinib with the cell cycle inhibitors (BI-2536, GSK-1070916 and paclitaxel or docetaxel) in the *MYC*-amplified cancer cell line NCI-H82. All combinations showed synergy. Neratinib with the Aurora inhibitor GSK-1070916 and neratinib with a taxane were further tested in three sets of cell lines with amplified or non-amplified *MYC*, from breast, colon and lung cancers (Figs 9, 10 and S4, S5 Figs).

Neratinib and GSK-1070916 show synergy in the *MYC*-amplified breast cancer line AU-565, but not in the wild-type control BT549 (Fig 9A and 9B). Therefore, in this context, neratinib and GSK-1070916 are a targeted combination. In colon and lung cancer cell lines, the combination shows general synergistic effects, and higher efficacy in *MYC*-amplified lung cancer cell lines (S4 Fig).

Neratinib and paclitaxel show synergy in the *MYC*-amplified lung cancer line NCI-H82, *i.e.* the combination induces a complete response, while the single agents show only a partial effect (Fig 10A). This is not the case in the non-amplified control A427 (Fig 10B). Combination of neratinib with another taxane, docetaxel, confirms this synergy in the same pair of cell lines, (S5 Fig). In colon cancer cell lines, there is a more general synergistic effect. In breast cancer cell lines, neratinib and docetaxel show a synergistic effect specific for the *MYC*-amplified line (S5 Fig). This indicates that the combination of neratinib and a taxane can be targeted, depending on tumor cell type.

Interestingly, the observed synergies for neratinib and taxanes are similar to those of the neratinib and GSK-1070916 combination (compare for instance Fig 9 with S5D and S5E Fig, or Fig 10 with S4C and S4D Fig).

Targeted combinations for *KRAS*-mutant cancer

The cell line profiling revealed that, among others, the ERBB2 kinase inhibitor TAK-165, the Aurora kinase inhibitor MLN-8054 and the MEK kinase inhibitor trametinib specifically inhibit proliferation of *KRAS*-mutant cell lines (Fig 7, Table 1). These findings are confirmed by the presence of other MEK inhibitors (PD-0325901 and AZD-6244) and another Aurora inhibitor (AMG-900) in the list of *KRAS*-mutant-targeted compounds (Fig 7). The activity of TAK-165 is not confirmed by other EGFR or ERBB2 inhibitors.

To search for targeted synergies, we combined the growth factor signaling inhibitor TAK-165 with an inhibitor of proliferation (trametinib) or a cell cycle inhibitor (MLN-8054) in proliferation assays with the *KRAS*-mutant A427 lung cancer cell line (Fig 11, S7 and S8 Figs). Particularly the combination of TAK-165 and trametinib shows synergy (Fig 11). This combination was further profiled in a *KRAS*-wild type lung cancer cell line (Fig 11B), and a pair of *KRAS*-mutant and wild type colon cancer cell lines (S7 Fig). Synergy appears in both lung cancer cell lines, but in none of the colon cancer cell lines (S7 Fig), indicating that the synergy of TAK-165 and trametinib depends on tissue type.

Discussion

Targeted synergy

Combination treatment is a cornerstone of cancer chemotherapy and there is great interest to identify new combinations *in vitro* to improve treatment outcome [1–6]. However, truly synergistic combinations are rare [4]. Here, we describe a novel work flow to study compound synergy. We applied this approach to validate a number of literature examples, and subsequently, used it to identify novel combinations of existing drugs that specifically target difficult-to-drug cancer genes. First, we compared curve shift analysis with combination matrix screening. We found that curve shift analysis is a more robust method than combination matrix screening with Bliss-scoring. The signal-to-noise ratios of synergistic and additive control experiments (Figs 1D, 2C and 2D) show that curve shift analysis has the least inter-assay variation. However, we want to point out that the $CI_{0.5}$ only takes one response level (50%) into account and that it remains important to visually inspect curve shape. The superiority of the curve shift method, compared to Bliss methods, most likely arises from its analysis of IC_{50}s, rather than maximum effects, which are more prone to variation.

The curve shift method confirms the synergistic activity of MEK inhibitors, such as trametinib, with PI3K inhibitors, such as GDC-0941 (Fig 1), and the synergistic activity of trametinib with the BRAF inhibitors dabrafenib and vemurafinib (Fig 3) [28]. Curve shift and CI analysis shows that trametinib is more synergistic with dabrafenib than with vemurafinib. This correlates with the higher selectivity of dabrafenib for *BRAF*-mutant cell lines in comparison to vemurafenib, which in turn is related to the higher biochemical potency of dabrafenib [31].

We also confirm the synergistic activity of BRAF and EGFR inhibitors in *BRAF*-mutant colon cancer cell lines (Fig 4) [29, 30].The claim that EGFR and BRAF inhibitors are inactive in *BRAF*-mutant colon cancer lines appears unfounded however [29, 30], as we observed a clear IC_{50} response (Fig 4). Interestingly, there is an increase in maximum efficacy of dabrafenib and vemurafenib induced by low concentrations of EGFR inhibitors (Fig 4). In combination with gefitinib, vemurafenib seems to be more synergistic than dabrafenib, which might be related to the better biochemical selectivity of vemurafenib [31]. The superiority of vemurafenib here

contrasts with the superiority of dabrafenib in combination with trametinib (Fig 3); it seems that both BRAF-dependent biological systems have their own optimal set of synergistic inhibitors.

We also used our setup to study if synergistic interactions are exclusive to (a specific subset of) cancer cells, and whether synergistic toxicities are absent in non-transformed cells, or in cells carrying a wild-type variant of the oncogene. The combination of the PI3K and MEK inhibitor not only shows synergy in cancer cells but also in non-transformed fibroblasts, a model for normal, non-malignant cells (Fig 1E). This is in line with the clinical observation that the combination has significant additional toxicity [40].

The combination of dabrafenib and trametinib is more specific in its synergistic interaction, as it only occurs in *BRAF*-mutant cell lines, not in *BRAF*-wild type cell lines (Fig 3). In fibroblasts the combination is antagonistic (S2 Fig). This is in line with the limited additional toxicity observed in combination treatment in the clinic [41]. This, and the subsequent FDA approval for dabrafenib with trametinib, supports the notion that synergistic combinations are interesting for follow-up, but that *targeted* synergistic combinations such as trametinib and dabrafenib are even more promising for further *in vivo* evaluation.

The example of dabrafenib and trametinib moreover shows that compounds that target the same subset of cancer cells (*i.e.*, *BRAF*-mutant) can act synergistically. We built on this notion in our selection of drug combinations to target *CTNNB1*, *KRAS* and *MYC*-driven cell growth.

Targeting *CTNNB1*-mutations

Mutations in the *CTNNB1* (β-catenin) gene affect the Wnt pathway and occur in a high percentage of uterine and colorectal cancers, and medullo-blastomas [42,43]. We identified two inhibitors that potently and significantly inhibit cells expressing mutant *CTNNB1*: trametinib and ICG-001 (Fig 6, Table 1). This suggests that these compounds might find an application for treatment of this cancer subtype. The targeting effect of trametinib is consistent with its role in the synthesis of β-catenin [44]; that of ICG-001 is consistent with its known inhibition of signaling downstream of β-catenin in the Wnt-pathway [39].

Subsequent combination studies revealed synergic interaction between trametinib and ICG-001 (Fig 8) in the *CTNNB1*-wild type SW620 colon cancer cell line and not in the *CTNNB1*-mutant colon cancer line SW48 (Fig 8). Although this seems counterintuitive, the SW620 line contains an *APC* mutation, and thereby is also dependent on Wnt signalling for growth (S2 Table). In fact, all colon cancer cell lines tested contain Wnt signalling mutations, though only in two, synergy is observed (S3 Fig). These (HCT-115 and SW620) are the only ones that have *TP53* mutations in addition to Wnt signalling mutations (S2 Table). It is known that wild type *TP53* can contribute to drug resistance in colon cancer cell lines [45] and cells with wild type TP53 were relatively resistant to trametinib in a profiling study in a 44-cell line panel [31]. Thus, in colon cancer, ICG-001 and trametinib might only show synergy in presence of dual Wnt-pathway and *TP53* mutations. In lung cancer cell lines, trametinib and ICG-001 show synergy in the cell line with a Wnt-pathway (*CTNNB1)* mutation and not in the wild type control (S3 Fig). Therefore, trametinib and ICG-001 are a synergistic drug pair in particular subsets of cell lines.

Targeting *MYC*-amplifications

Amplification of *MYC* occurs in many cancers, including more than 40% of ovarian cancers [42,43]. Despite being one of the most investigated oncogenes, there are currently no therapies that target MYC signaling specifically [21, 22]. We discovered that a number of Aurora and PLK1 kinase inhibitors were relatively selective for *MYC*-amplified cells, as well as paclitaxel,

docetaxel and neratinib (Table 1). Since GSK-1070916 and BI-2536 belong to the most selective kinase inhibitors developed so far [46], it appears that indeed Aurora B/C and PLK1 inhibition are responsible for the selective targeting of *MYC*-amplified cells. These results agree with RNAi-based synthetic lethality studies, which have identified Aurora B inhibition as synthetically lethal with *MYC*-driven cell growth [20, 47].

Neratinib is a spectrum-selective EGFR/ERBB2 inhibitor [48]. The presence of other highly selective EGFR/ERBB2 inhibitors such as afatinib and lapatinib among the list of *MYC*-targeting agents (Fig 7), and the absence of selective EGFR inhibitors, such as gefitinib and erlotinib, suggests that ERBB2 inhibition, or inhibition of both EGFR and ERBB2 is responsible for *MYC*-targeting. This is supported by recent *in vitro* and *in vivo* studies that show that *ERBB2* and *MYC* can cooperate to induce aggressiveness in breast cancer [49].

Subsequent combination studies showed synergistic interaction between neratinib and GSK-1070916 in a variety of cancer cell lines. The results in breast cancer lines (Fig 9) show that neratinib and GSK-1070916 can be a targeted combination in the context of *MYC* amplification. Other groups have also found synergy between EGFR/ERBB2 and Aurora inhibition, either in genomic or pharmacological studies [50–53]. However, the synergy in context of *MYC*-amplification has not been described before, to our knowledge. Interestingly, the AU-565 cell line that shows the best synergy (Fig 9A) also has an *ERBB2* amplification in addition to *MYC* (S2 Table). It is known that MYC is a downstream effector of ERBB2 signaling [54], that *MYC*-amplification results in Aurora kinase- dependent growth [47], and that co-amplification of *ERBB2* and *MYC* is a frequent event in cancer [55]. Therefore, we propose that *ERBB2*, *MYC* and Aurora form an integrated oncogenic signaling network that is optimally inhibited by the combination of neratinib and GSK-1070916 in *MYC*-amplified cells.

A second combination that shows promise is that of neratinib and taxanes such as paclitaxel and docetaxel. This combination also shows MYC-dependent synergistic interaction in lung cancer and breast cancer cell lines (Fig 10 and S5 Fig), similar to the combination of neratinib and GSK-1070916. This might be because both taxanes and the Aurora inhibitor GSK-1070916 impact cell cycle progression. In general, the synergistic interactions are less strong for the taxane combinations. The combination of neratinib and paclitaxel is currently tested in the clinic for treatment of breast cancer [56]. Our data support further experiments with this combination in other indications.

Targeting *KRAS*-mutations

KRAS activation occurs in more than 70% of pancreatic cancers and the identification of specific inhibitors or combinations that inhibit KRAS signaling has proven to be difficult [57]. Our screen identified three types of kinase inhibitors that target *KRAS*-driven cell growth: various MEK inhibitors, including trametinib, the ERBB2 inhibitor TAK-165, and Aurora kinase inhibitors, such as MLN-8054 and AMG-900 (Fig 7). MEK is part of the RAS pathway and it has been shown before that *RAS*-mutant cell lines are sensitive to MEK inhibitors [36]. Aurora kinases have never been implicated in targeting of *KRAS*-mutations, despite a wealth of synthetic lethal studies [20, 21, 57, 58]. MLN-8054 is a selective Aurora A inhibitor [48], AMG-900 has pan-Aurora activity [48]. This, and the absence of the selective Aurora B/C inhibitor GSK-1070916 in the list, suggests that the selective *KRAS*-targeting is due to inhibition of Aurora A. This is further supported by data on the Aurora A/B inhibitor ZM-447439, which shows significantly more potent IC_{50}s on *KRAS*-mutant cell lines in a 1000-cell line screen of the Sanger centre [9].

TAK-165 [59] is the only growth factor signaling inhibitor in our screen that shows *KRAS*-targeting. Interestingly, it is also the most *KRAS*-targeted compound (Fig 7). Recently, it was

described that ERBB2 and ERBB3 signaling play an important role in *KRAS*-dependent cell growth [29, 60]. It is, however, unclear why TAK-165 is so particularly adept at targeting *KRAS*, while other inhibitors that inhibit ERBB2, such neratinib, lapatinib or afatinib, are not. TAK-165 distinguishes itself from these other inhibitors in that it does not inhibit EGFR [46, 59]. We conclude that inhibition of ERBB2 without simultaneous inhibition of EGFR might contribute to targeting *KRAS*-mutant cell lines.

Combination studies reveal trametinib and TAK-165 as a synergistic pair particularly in lung cancer cell lines (Fig 11). The biological significance of this combination is supported by the recent results of Sun *et al.*, who showed that a MEK and ERBB2 inhibitor act synergistically in *KRAS*-mutant cell lines [60]. In comparison with this work, our study explored a broader range of inhibitors, and we identified TAK-165 and trametinib as the best pair to take forward. Because both compounds have been already tested in humans, they provide excellent candidates for validating the concept of dual inhibition of MEK and ERBB2 in more advanced models of *KRAS*-mutant cancer.

Conclusion

We have developed a workflow to identify synergistic combinations based on combining compounds that target the same cancer driver genes or pathways. In agreement with earlier, large scale combination studies, our experience is that truly synergistic combinations are rare. For instance, Borisy *et al.* who screened 100,000 combinations identified forty-one synergistic combinations (0.04%) [4]. Cokol *et al.* who screened two hundred combinations identified thirty-eight (19%) [5]. Using our rational approach, that combines compounds that target similar cancer cell line subsets, we discovered six novel synergistic combinations, of eight tested, a success rate of 75%. Despite the limited experience with this approach, it seems that compounds that are cellularly more selective also give rise to more potent synergies. A good example is dabrafenib, which is more cellularly selective for cells that grow dependent on BRAF compared to vemurafenib [31], and is also the superior partner of the MEK inhibitor trametinib. For PI3K inhibitors in combination with MEK inhibitors, we have also observed that the most cellularly selective inhibitors result in the most potent synergy. However, there are also exceptions, such as our study of EGFR and BRAF-inhibitors shows, in which vemurafenib is superior to dabrafenib. The new compound combinations that we have discovered are worth further study. All compounds in the suggested combinations have already been in clinical trials—except ICG-001- and therefore can rapidly progress to further *in vivo* validation. The generation of increasingly sophisticated targeted combinations, assembled on the basis of *in vitro* data, will be an important step in overcoming efficacy and resistance problems associated with targeted single agent therapy.

Supporting Information

S1 Table. Sources of inhibitors used in the cell line profiling
(XLSX)

S2 Table. Overview of the cell lines in the Oncolines cell panel and presence of twenty-three commonly occurring cancer genes
(XLSX)

S3 Table. IC_{50}s of compounds studied in synergy experiments in forty-four cell line Oncolines panel
(XLSX)

S4 Table. CI values of all compound combinations tested
(XLSX)

S1 Equation. Formula for Bliss parameterfitting
(PDF)

S1 Fig. Curve shift experiment of the combination of GDC-0941 and AZD-6244 in the hTERT immortalized cell line BJ5-ta
(PDF)

S2 Fig. Curve shift experiment of the combination of trametinib and dabrafenib in the hTERT immortalized cell lines BJ5-ta and RPE-1
(PDF)

S3 Fig. Curve shift experiments of the combination of ICG-001 and trametinib
(PDF)

S4 Fig. Curve shift experiments of the combination of neratinib (HKI-272) and GSK-1070916
(PDF)

S5 Fig. Curve shift experiments of the combination of neratinib (HKI-272) and docetaxel
(PDF)

S6 Fig. Curve shift experiments of the combination of neratinib (HKI-272) and BI-2536
(PDF)

S7 Fig. Curve shift experiments of the combination of trametinib and TAK-165
(PDF)

S8 Fig. Curve shift experiments of the combination of MLN-8054 and TAK-165
(PDF)

Author Contributions

Conceived and designed the experiments: JCMU JADMR RCB GJRZ. Performed the experiments: JADMR AMVD MBWP JAPSH JRFV. Analyzed the data: JCMU JADMR JAPSH RCB GJRZ. Contributed reagents/materials/analysis tools: JDM. Wrote the paper: JCMU GJRZ.

References

1. Al-Lazikani B, Banerji U, Workman P (2012) Combinatorial drug therapy for cancer in the post-genomic era. Nature Biotech 30: 679–682.
2. Yap TA, Omlin A, de Bono JS (2013) Development of therapeutic combinations targeting major cancer signaling pathways. J Clinical Invest 20: 1592–1605.
3. Li F, Zhao C, Wang L (2014) Molecular-targeted agents combination therapy for cancer: developments and potentials. Int J Cancer 134: 1257–1269. doi: 10.1002/ijc.28261 PMID: 23649791
4. Borisy AA, Elliott PJ, Hurst NW, Lee MS, Lehár J, Price ER, et al. (2003) Systematic discovery of multicomponent therapeutics. Proc Natl Acad Sci USA; 100: 7977–7982. PMID: 12799470
5. Cokol M, Chua HN, Tasan M, Mutlu B, Weinstein ZB, Suzuki Y, et al. (2011) Systematic exploration of synergistic drug pairs. Molecular systems biology 7: 544. doi: 10.1038/msb.2011.71 PMID: 22068327
6. Mathews Griner LA, Guha R, Shinn P, Young RM, Keller JM, Liu D, et al. (2014) High throughput combinatorial screening identifies drugs that cooperate with ibrutinib to kill activated B-cell-like diffuse large B-cell lymphoma cells. Proc Natl Acad Sci USA 111: 2349–2354. doi: 10.1073/pnas.1311846111 PMID: 24469833
7. Zhao L, Wientjes MG, Au JLS (2004) Evaluation of combination chemotherapy: integration of nonlinear regression, curve shift, isobologram and combination index analyses. Clin Canc Res 10: 7994–8004.

8. Chou T-C (2010) Drug combination studies and their synergy quantification using the Chou-Talalay method. Cancer Res 70: 440–446. doi: 10.1158/0008-5472.CAN-09-1947 PMID: 20068163
9. Garnett MJ, Edelman EJ, Heidorn SJ, Greenman CD, Dastur A, Lau KW, et al. (2012) Systematic identification of genomic markers of drug sensitivity in cancer cells. Nature 483: 570–575. doi: 10.1038/nature11005 PMID: 22460902
10. Barretina J, Caponigro G, Stransky N, Venkatesan K, Margolin AA, Kim S, et al. (2013) The Cancer Cell Line Encyclopedia enables predictive modelling of anticancer drug sensitivity. Nature 483: 603–607.
11. Druker BJ, Talpaz M, Resta DJ, Peng B, Buchdunger E (2001) Efficacy and safety of a specific inhibitor of the BCR-ABL tyrosine kinase in chronic myeloid leukemia. N Engl J Med 344: 1031–1037. PMID: 11287972
12. Hauschild A, Grob JJ, Demidov LV, Jouary T, Gutzmer R, Millward M, et al. (2012) Dabrafenib in BRAF-mutated metastatic melanoma: a multicentre, open label, phase 3 randomised trial. The Lancet 380: 358–365. doi: 10.1016/S0140-6736(12)60868-X PMID: 22735384
13. Chapman PB, Hauschild A, Robert C, Haanen JB, Ascierto P, Larkin J, et al. (2011) Improved survival with vemurafenib in melanoma with BRAF V600E mutation. N Engl J Med 364: 2507–2516. doi: 10.1056/NEJMoa1103782 PMID: 21639808
14. Holohan C, van Schaeybroeck S, Longley DB, Johnston PG (2013) Cancer drug resistance: an evolving paradigm. Nature Rev Cancer 13: 714–726. doi: 10.1038/nrc3599 PMID: 24060863
15. Hopkins AL, Groom CR (2002) The druggable genome. Nature Rev Drug Disc 1: 727–730. PMID: 12209152
16. Chan DA, Giaccia AJ (2011) Harnessing synthetic lethal interactions in anticancer drug discovery. Nature Rev Drug Disc 10: 351–364. doi: 10.1038/nrd3374 PMID: 21532565
17. McLornan DP, List A, Mufti GJ (2014) Applying synthetic lethality for the selective targeting of cancer. New Engl J Med 371: 1725–1735. doi: 10.1056/NEJMra1407390 PMID: 25354106
18. Barbie DA, Tamayo P, Boehm JS, Kim SY, Moody SE, Dunn IF, et al. (2009) Systematic RNA interference reveals that oncogenic KRAS-driven cancers require TBK1. Nature 462: 108–112. doi: 10.1038/nature08460 PMID: 19847166
19. Scholl C, Fröhling S, Dunn IF, Schinzel AC, Barbie DA, Kim SY, et al. (2009) Synthetic lethal interaction between oncogenic KRAS dependency and STK33 suppression in human cancer cells. Cell 137: 821–834. doi: 10.1016/j.cell.2009.03.017 PMID: 19490892
20. Yang D, Liu H, Goga A, Kim S, Yuneva M, Bishop JM (2010) Therapeutic potential of a synthetic lethal interaction between the MYC proto-oncogene and inhibition of aurora-B kinase Proc Natl Acad Sci USA 107: 13836–13841. doi: 10.1073/pnas.1008366107 PMID: 20643922
21. Toyoshima M, Howie HL, Imakura M, Walsh RM, Annis JE, Chang AN, et al. (2012) Functional genomics identifies therapeutic targets of MYC-driven cancer. Proc Natl Acad Sci USA 109: 9545–9550. doi: 10.1073/pnas.1121119109 PMID: 22623531
22. Evan G (2012) Cancer. Taking a back door to target MYC. Science 335: 293–294. doi: 10.1126/science.1217819 PMID: 22267799
23. Naik S, Dothager RS, Marasa J, Lewis CL, Piwnica-Worms D (2009) Vascular endothelial growth factor receptor-1 is synthetic lethal to aberrant β-catenin activation in colon cancer. Clin Cancer Res 15: 7529–7537. PMID: 20008853
24. Kim SY, Dunn IF, Firestein R, Gupta P, Wardwell L, Repich K, et al. (2010) CK1epsilon is required for breast cancers dependent on β-catenin activity. PLOS ONE 5: e8979. doi: 10.1371/journal.pone.0008979 PMID: 20126544
25. Babij C, Zhang Y, Kurzeja RJ, Munzli A, Shehabeldin A, Fernando M, et al. (2011) STK33 kinase activity is nonessential in KRAS-dependent cancer cells. Cancer Res 71: 5818–5826. doi: 10.1158/0008-5472.CAN-11-0778 PMID: 21742770
26. Sos ML, Fischer S, Ullrich R, Peifer M, Heuckmann JM, Koker M, et al. (2009) Identifying genotype-dependent efficacy of single and combined PI3K- and MAPK-pathway inhibition in cancer. Proc Natl Acad Sci USA 104: 18351–18356.
27. Liu D, Xing M (2008) Potent inhibition of thyroid cancer cells by the MEK inhibitor PD0325901 and its potentiation by suppression of the PI3K and NFκB pathways. Thyroid 18: 853–864. doi: 10.1089/thy.2007.0357 PMID: 18651802
28. King AJ, Arnone MR, Bleam MR, Moss KG, Yang J, Fedorowicz KE, et al. (2013) Dabrafenib; preclinical characterisation, increased efficacy when combined with trametinib, while BRAF/MEK tool combination reduced skin lesions. PLOS ONE 8: e67583. doi: 10.1371/journal.pone.0067583 PMID: 23844038

29. Prahallad A, Sun C, Huang S, Nicolantonio F, Salazar R, Zecchin D, et al. (2012) Unresponsiveness of colon cancer to BRAF(V600E) inhibition through feedback activation of EGFR. Nature 483: 100–103. doi: 10.1038/nature10868 PMID: 22281684

30. Corcoran RB, Ebi H, Turke AB, Coffee EM, Nishino M, Cogdill AP, Brown RD, et al. (2012) EGFR mediated reactivation of MAPK signaling contributes to insensitivity of BRAF-mutant colorectal cancers to RAF inhibition with vemurafenib. Cancer Discovery 2: 227–235. doi: 10.1158/2159-8290.CD-11-0341 PMID: 22448344

31. Uitdehaag JCM, de Roos JADM, van Doornmalen AM, Prinsen MBW, de Man J, Tanizawa Y, et al. (2014) Comparison of the cancer gene targeting and biochemical selectivities of all targeted kinase inhibitors approved for clinical use. PLOS ONE 9: e92146. doi: 10.1371/journal.pone.0092146 PMID: 24651269

32. Straetemans R, O'Brien T, Wouters L, van Dun J, Janicot M, Bijnens L, et al. (2005) Design and analysis of drug combination experiments. Biometrical J 47: 299–308. PMID: 16053254

33. Bliss CI (1939) The toxicity of poisons applied jointly. Annals of applied biology 26: 585–615.

34. Uitdehaag JCM, Zaman GJR (2011) A theoretical entropy score as a single value to express inhibitor selectivity. BMC Bioinformatics 12: 94. doi: 10.1186/1471-2105-12-94 PMID: 21486481

35. R development core team (2008) R: a language and environment for statistical computing. R Foundation for statistical computing, Vienna, Austria

36. Solit DB, Garraway LA, Pratilas CA, Sawai A, Getz G, Basso A, et al. (2006) BRAF mutation predicts sensitivity to MEK inhibition. Nature 439: 358–362. PMID: 16273091

37. Loewe S, Muischnek H (1926) Über kombinationswirkungen. Arch Exp Pathol Pharmakol 114: 313–326.

38. Haagensen EJ, Kyle S, Beale GS, Maxwell RJ, Newell DR (2012) The synergistic interaction of MEK and PI3K inhibitors is modulated by mTOR inhibition. Br J Cancer 106: 1386–1394. doi: 10.1038/bjc.2012.70 PMID: 22415236

39. Emami KH, Nguyen C, Ma H, Kim DH, Jeong KW, Eguchi M, et al. (2004) A small molecule inhibitor of ß-catenin/cyclic AMP response element-binding protein transcription. Proc Natl Acad Sci USA 101: 12682–12687. PMID: 15314234

40. Shimizu T, Tolcher AW, Papadopoulos KP, Beeram M, Rasco DW, Smith LS, et al. (2012) The clinical effect of the dual-targeting strategy involving PI3K/AKT/mTOR and RAS/MEK/ERK pathways in patients with advanced cancer. Clin Cancer Res 18: 2316–2325. doi: 10.1158/1078-0432.CCR-11-2381 PMID: 22261800

41. Flaherty KT, Infante JR, Daud A, Gonzalez R, Kefford RF, Sosman J, et al. (2012) Combined BRAF and MEK inhibition in melanoma with BRAF V600 mutations. New Engl J Med 367: 1694–1703. doi: 10.1056/NEJMoa1210093 PMID: 23020132

42. The Cancer Genome Atlas. Available: http://www.cancergenome.nih.gov. Accessed 26 November 2014.

43. Cerami E, Gao J, Dogrusoz U, Gross BE, Sumer SO, Aksoy BA, et al. (2012) The cBio cancer genomics portal: an open platform for exploring multidimensional cancer genomics data. Cancer Discovery 2: 401–404. doi: 10.1158/2159-8290.CD-12-0095 PMID: 22588877

44. Gosens R, Baarsma HA, Heijink IH, Oenema TA, Halayko AJ, Meurs H, et al. (2010) De novo stabilization of β-catenin via H-Ras and MEK regulates airway smooth muscle growth. The FASEB J 24: 757–768. doi: 10.1096/fj.09-136325 PMID: 19906679

45. Violette S, Poulain L, Dussaulx E, Pepin D, Faussat AM, Chambaz J, et al. (2002) Resistance of colon cancer cells to long-term 5-fluorouracil exposure is correlated to the relative level of Bcl-2 and Bcl-X_L in addition to *BAX* and *P53* status. Int J Cancer 98: 498–504. PMID: 11920608

46. Uitdehaag JCM, Verkaar FA, Alwan H, de Man J, Buijsman RC, Zaman GJ (2012) A guide to picking the most selective kinase inhibitor tool compounds for pharmacological validation of drug targets. Br J Pharmacol 166: 858–876. doi: 10.1111/j.1476-5381.2012.01859.x PMID: 22250956

47. den Hollander J, Rimpi S, Doherty JR, Rudelius M, Buck A, Hoellein A, et al. (2010) Aurora kinases A and B are up-regulated by Myc and are essential for maintenance of the malignant state. Blood 116: 1498–1505. doi: 10.1182/blood-2009-11-251074 PMID: 20519624

48. Davis MI, Hunt JP, Herrgard S, Ciceri P, Wodicka LM, Pallares G, et al. (2011) Comprehensive analysis of kinase inhibitor selectivity. Nature Biotech 29: 1046–1051. doi: 10.1038/nbt.1990 PMID: 22037378

49. Nair R, Roden DL, Teo WS, McFarland A, Junankar S, Ye S, et al. (2014) cMYC and HER cooperate to drive a stem-like phenotype with poor prognosis in breast cancer. Oncogene 33: 3992–4000. doi: 10.1038/onc.2013.368 PMID: 24056965

50. Hung LY, Tseng JT, Lee YC, Xia W, Wang YN, Wu ML, Chuang YH, et al. (2008) Nuclear epidermal growth factor receptor (EGFR) interacts with signal transducer and activator of transcription 5 (STAT5)

in activating *Aurora-A* gene expression. Nucleic Acids Res 36: 4337–4351. doi: 10.1093/nar/gkn417 PMID: 18586824

51. Addepalli MK, Ray KB, Kumar B, Ramnath RL, Chile S, et al. (2010) RNAi-mediated knockdown of AURKB and EGFR shows enhanced therapeutic efficacy in prostate tumor regression. Gene Therapy 17: 352–359. doi: 10.1038/gt.2009.155 PMID: 19956271
52. Astsaturov I, Ratushny V, Sukhanova A, Einarson MB, Bagnyukova T, Zhou Y, et al. (2010) Synthetic lethal screen of an EGFR-centered network to improve targeted therapies. Sci Signal 3: 140.
53. Hoellein A, Pickhard A, von Keitz F, Schoeffmann S, Piontek G, Rudelius M, et al. (2010) Aurora kinase inhibition overcomes cetuximab resistance in squamous cell cancer of the head and neck. Oncotarget 2: 599–609.
54. Hynes NE, Lane HA (2001) Myc and mammary cancer: Myc is a downstream effector of the ERBB2 receptor tyrosine kinase. J Mammary Gland Biol Neoplasia 6: 141–150. PMID: 11467449
55. Mitsui F, Dobashi Y, Imoto I, Inazawa J, Kono K, Fujii H, et al. (2007) Non-incidental coamplification of *Myc* and *ERBB2*, and *Myc* and *EGFR*, in gastric adenocarcinomas. Modern Pathology 20: 622–631. PMID: 17431415
56. Chow LWC, Xu B, Gupta S, Freyman A, Zhao Y, Abbas R, Vo Van ML, Bondarenko I (2013) Combination neratinib (HKI-272) and paclitaxel therapy in patients with HER2-positive metastatic breast cancer. Br J Cancer 108: 1985–1993. doi: 10.1038/bjc.2013.178 PMID: 23632474
57. Vasan N, Boyer JL, Herbst RS (2014) A RAS renaissance: emerging targeted therapies for KRAS-mutated non-small cell lung cancer. Clin Cancer Res 20: 3921–3930. doi: 10.1158/1078-0432.CCR-13-1762 PMID: 24893629
58. Singh A, Settleman J (2009) Oncogenic K-ras "addiction" and synthetic lethality. Cell cycle 8: 2676–2677. PMID: 19690457
59. Nagasawa J, Mizokami A, Koshida K, Yoshida S, Naito K, et al. (2006) Novel HER2 selective tyrosine kinase inhibitor TAK-165 inhibits bladder, kidney and androgen-dependent prostate cancer *in vitro* and *in vivo*. Int J Urol 13: 587–592. PMID: 16771730
60. Sun C, Hobor S, Bertotti A, Zecchin D, Huang S, Galimi F, et al. (2014) Intrinsic resistance to MEK inhibition in KRAS mutant lung and colon cancer through transcriptional induction of ERBB3. Cell Rep 7: 86–93. doi: 10.1016/j.celrep.2014.02.045 PMID: 24685132
61. Rabindran SK, Discafani CM, Rosfjord EC, Baxter M, Floyd MB, Golas J, et al. (2004) Antitumor activity of HKI-272, an orally active, irreversible inhibitor of the HER-2 tyrosine kinase. Cancer Res 64: 3958–3965. PMID: 15173008
62. Adams ND, Adams JL, Burgess JL, Chaudhari AM, Copeland RA, Donatelli CA, et al. (2010) Discovery of GSK-1070916, a potent and selective inhibitor of Aurora B/C kinase. J Med Chem 27: 3973–4001.
